# De novo leaf and root transcriptome analysis to explore biosynthetic pathway of Celangulin V in *Celastrus angulatus* maxim

**DOI:** 10.1186/s12864-018-5397-z

**Published:** 2019-01-05

**Authors:** Weiguo Li, Ranran Xu, Xiaoguang Yan, Dongmei Liang, Lei Zhang, Xiaoyu Qin, Qinggele Caiyin, Guangrong Zhao, Wenhai Xiao, Zhaonong Hu, Jianjun Qiao

**Affiliations:** 1 Department of Pharmaceutical Engineering, School of Chemical Engineering and Technology, Tianjin University, Tianjin, 300072 People’s Republic of China; 2 Key Laboratory of Systems Bioengineering (Ministry of Education), Tianjin University, Tianjin, 300072 People’s Republic of China; 3 SynBio Research Platform, Collaborative Innovation Center of Chemical Science and Engineering (Tianjin), Tianjin, 300072 People’s Republic of China; 4 College of Plant Protection, Institute of Pesticide Science, Northwest A&F University, Yangling, Shaanxi 712100 People’s Republic of China; 5 Key Laboratory of Botanical Pesticide R&D in Shaanxi Province, Yangling, Shaanxi 712100 People’s Republic of China

**Keywords:** *Celastrus angulatus* maxim, Celangulin V, Natural insecticides, Secondary metabolism, Sesquiterpene biosynthesis, Transcriptome

## Abstract

**Background:**

*Celastrus angulatus* Maxim is a kind of crucial and traditional insecticidal plant widely distributed in the mountains of southwest China. Celangulin V is the efficient insecticidal sesquiterpenoid of *C. angulatus* and widely used in pest control in China, but the low yield and discontinuous supply impeded its further popularization and application. Fortunately, the development of synthetic biology provided an opportunity for sustainable supply of Celangulin V, for which understanding its biosynthetic pathway is indispensable.

**Results:**

In this study, six cDNA libraries were prepared from leaf and root of *C. angulatus* before global transcriptome analyses using the BGISEQ-500 platform. A total of 104,950 unigenes were finally obtained with an average length of 1200 bp in six transcriptome databases of *C. angulatus*, in which 51,817 unigenes classified into 25 KOG classifications, 39,866 unigenes categorized into 55 GO functional groups, and 48,810 unigenes assigned to 135 KEGG pathways, 145 of which were putative biosynthetic genes of sesquiterpenoid and triterpenoid. 16 unigenes were speculated to be related to Celangulin V biosynthesis. De novo assembled sequences were verified by Quantitative Real-Time PCR (qRT-PCR) analysis.

**Conclusions:**

This study is the first report on transcriptome analysis of *C. angulatus*, and 16 unigenes probably involved in the biosynthesis of Celangulin V were finally collected. The transcriptome data will make great contributions to research for this specific insecticidal plant and the further gene mining for biosynthesis of Celangulin V and other sesquiterpene polyol esters.

**Electronic supplementary material:**

The online version of this article (10.1186/s12864-018-5397-z) contains supplementary material, which is available to authorized users.

## Background

With the increase of global population, natural insecticides become a trend of pesticide development for their environmental friendliness. *Celastrus angulatus* Maxim*,* extensively distributed in the mountains of southwest China, have been exploited as natural insecticide resource and folk medicines attributed to their active ingredients, including sesquiterpenes (β-agarofurans), alkaloids, and flavonoids [1]. Celangulin V, the major insecticidal ingredient of *C. angulatus,*, exhibits excellent activities against several insect species like *Pieris rapae (Lepidoptera)* and *Locusta migratoria manilensis (Orthoptera)* [1, 2] targeting on Na^+^/K^+^-ATPase (sodium/potassium pump) of these insects. As precisely identified, Celangulin V is a kind of sesquiterpene polyol esters with β-dihydroagarofuran skeleton, holding seven hydroxyl groups [1]. In the past 20 years, a series of Celangulin V derivates have been isolated by using bioassay-directed fractionation. A total of 44 bioactive compounds were firstly reported, displaying antifeedant, narcotic or toxic effects against some important agricultural pests [3, 4].

**Fig. 1:**
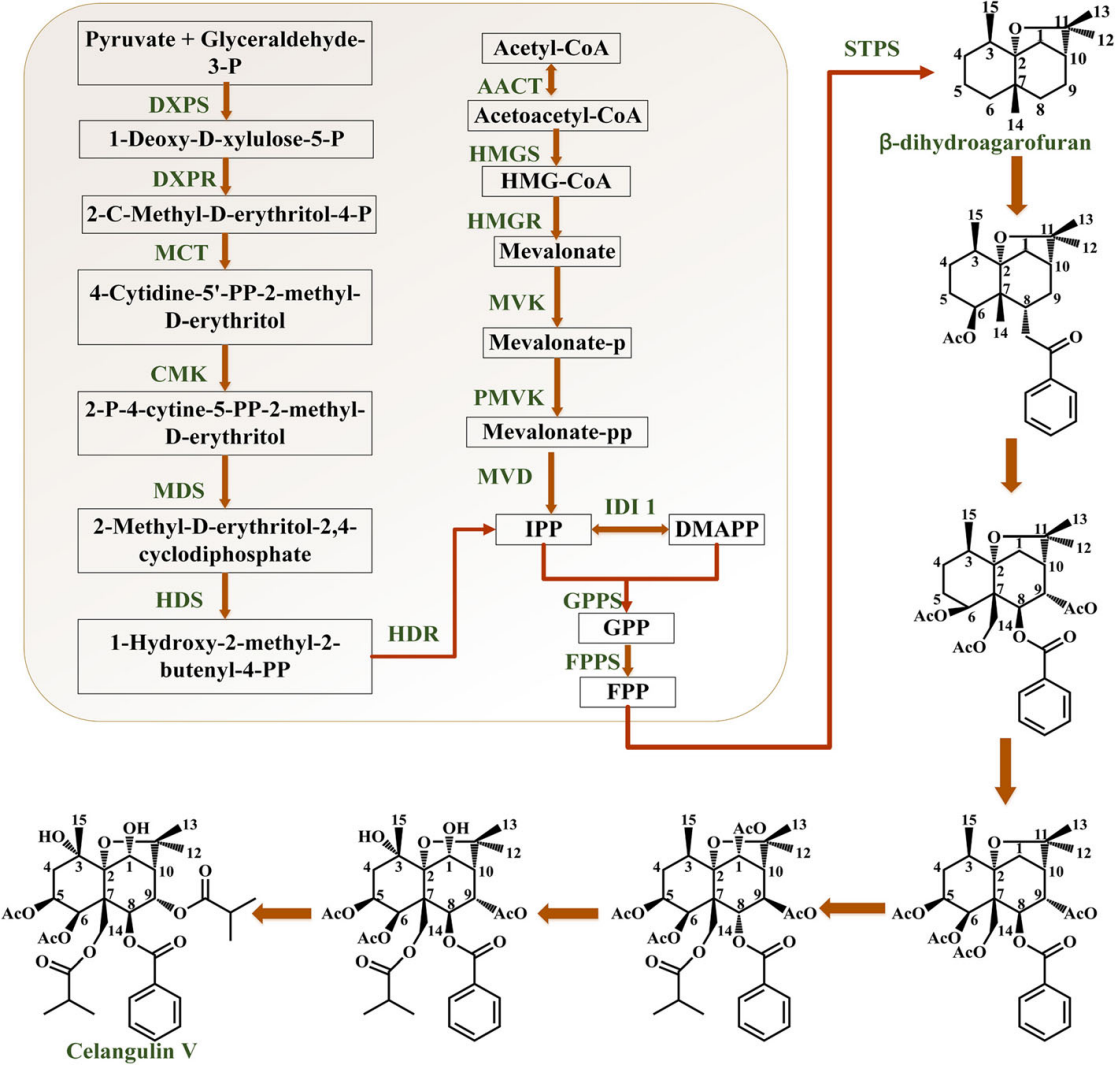
Putative Celangulin V biosynthetic pathway in *C. angulatus*. 1-deoxy-D-xylulose-5-phosphate synthase (DXPS); 1-deoxy-D-xylulose-5-phosphate reductoisomerase (DXPR); 2-C-methyl-D-erythritol 4-phosphate cytidylyltransferase (MCT); 4-(cytidine 5′-diphospho)-2-C-methyl-D-erythritol kinase (CMK); 2-C-methyl- D -erythritol 2,4-cyclodiphosphate synthase (MDS); (E)-4-hydroxy-3-methylbut-2-enyl diphosphate synthase (HDS); (E)-4-hydroxy-3-methylbut-2-enyl diphosphatereductase (HDR); C-acetyltransferase (AACT); Hydroxymethylglutaryl-CoA synthase (HMGS); Hydroxymethylglutaryl-CoA reductase (HMGR); Mevalonate kinase (MVK); Phosphomevalonate kinase (PMVK); mevalonate diphosphate decarboxylase (MVD); isopentenyl-diphosphate δ-isomerase (IDI); geranyl diphosphate synthase (GPPS); Farnesyl diphosphate synthase (FPPS). This figure was generated with Microsoft Visio Professional 2013

Celangulin V can be extracted from roots and leaves of *C. angulatus*, and the content in roots is higher than that in leaves [5–7]. The current supply is dependent on filed-grown plant with unstable yield. To address such problems, attempts have been made to produce Celangulin V in more eco-friendly means of biosynthesis. In general, the biosynthetic pathway of sesquiterpenes could be divided into three parts. Part I is isopentenyl diphosphate (IPP) and dimethylallyl pyrophosphate (DMAPP) biosynthesis [8, 9]. Part II is carbo- cyclization of sesquiterpene biosynthesis. Part III is the modification pathway of complex functional groups in the sesquiterpene skeleton [10–13]. The whole reaction is catalyzed by a large variety of enzymes with substrate specificities (Fig. 1).

So far, the researches of *C. angulatus* mainly focused on its phytochemical properties, including the extraction of active ingredients, the identification of active substances and the evaluation of insecticidal activity [14]. As the most important bioactive component of *C. angulatus*, Celangulin V was also well studied in structures and physicochemical properties [15, 16]. However, its biosynthetic and metabolic pathway remained poorly investigated, mainly owing to the absence of genomic or transcriptomic resources for this non-model species. Although no gene sequence information is available in public databases, we completed the transcriptome sequencing and analysis referring to the genome information of plants closely related to *C. angulatus*. Thus, comprehensive genomic information can be further analyzed for gene expression, molecular mechanisms and biological pathways [17, 18]. This strategy has been efficiently used in traditional Chinese medicinal species such as *Gardenia jasminoides* [19], *Pseudostellaria heterophylla* [20], *Panax notoginseng* [21] and *Cephalotaxus hainanensis* [22].

In this study, functional genes involved in sesquiterpene biosynthesis in *C. angulatus* were screened using de novo transcriptome sequencing. Four unigenes encoding sesquiterpene synthases, eight unigenes encoding cytochrome P450s (CYP450s) and four unigenes encoding acyltransferases were identified from Leaf and Root transcription databases of *C. angulatus* through BGISEQ-500. The candidate transcript sequences were listed in the Additional file 1. Differentially expressed genes (DEGs) were also analyzed. Such databases could be used as an important resource to investigate the biosynthetic pathway of Celangulin V in *C. angulatus*. Furthermore, this database will supply important clues to explore biological characteristics genetically in other plants with close relationship to *C. angulatus*.

## Methods

### Plant materials

Leaf and root tissues of *C. angulatus* were randomly picked from the exposition park of Northwest A&F University, followed by transcriptome analysis on May 11, 2017. Leaf and root samples were harvested from three plants for RNA extraction and three biological replicates (Leaf_1 and Root_1, Leaf_2 and Root_2, Leaf_3 and Root_3) were performed. Tissues were rinsed in water, cut into small pieces, frozen in liquid nitrogen immediately, and stored at − 80 °C for further analyses. The *C. angulatus* was authenticated by Prof. Wenjun Wu and Prof. Zhaonong Hu.

### cDNA library preparation and BGISEQ-500 sequencing for transcriptome analysis

Total RNA was extracted using an RNA plant Plus Reagent (Tiangen, Beijing, China) according to the manufacturer’s protocol. The extracted RNA was checked using a NanoDrop 2000 (Thermo, CA, USA), and the RNA concentration and integrity were assessed using the RNA Nano 6000 Assay Kit of the Agilent Bioanalyzer 2100 system (Agilent, CA, USA) to ensure that the RNA Integrity Number (RIN) values were above 7.0.

Oligo (dT) beads were used to isolate poly(A) + mRNA, which was fragmented to 250 bp. Fragmentation of the RNA and reverse transcription of double-strand cDNA (ds cDNA) by N6 random primer. The synthesized cDNA was subjected to end-repair and then was 3′ adenylated. Adaptors were ligated to the ends of these 3′ adenylated cDNA fragments; The ligation products were purified and many rounds of PCR amplification were performed to enrich the purified cDNA template using PCR primer; Denature the PCR product by heat and the single strand DNA is cyclized by splint oligo and DNA ligase; Each cDNA library was sequenced in a single lane of the BGISEQ-500 system with paired-end sequencing length of 100 bp according to the manufacturer’s instructions at the Beijing Genomics Institute (BGI-Shenzhen, China). The amounts of reads generated per sample was 10–11 Gb to obtain deep coverage of transcripts for de novo assembly.

### De novo assembly and functional annotation analysis of BGISEQ-500 sequencing

To obtain high-quality clean read data for de novo assembly, the raw reads from BGISEQ-500 were filtered by discarding the reads with adaptor sequences, reads with ambiguous “N” bases larger than 5% and removing the low-quality reads in which more than 20% bases had a Q-value < 15. All the downstream analyses were based on the resulting clean reads. We used the software Trinity (v2.0.6) [23], which was efficient to form contigs in reconstructing full-length transcripts across a broad range of expression levels and sequencing depths, with default parameters and a minimum contig length of 150 bp for assembly generation. These contigs were then further processed with sequence clustering software TGICL (v2.0.6) [24] to remove the redundant Trinity generated contigs.

To determine the functional annotation of the unigenes, a BLASTx search was performed with an E-value of 10^− 5^ against protein databases, including Nr (non-redundant) protein database, SwissPort, KOG (euKaryotic Orthologous Group database), and KEGG (Kyoto Encyclopedia of Genes and Genomes protein database). Besides, a BLASTn search was also performed against the Nt database. With Nr annotation, the Blast2GO [25] and InterProScan5 program was used to obtain the GO (Gene ontology) and InterPro annotation of unigenes, respectively. GO classification was then performed using WEGO software [26] to illustrate the distribution of gene functions including Biological Process, Cellular Component and Molecular Function.

### Differentially expressed unigene analysis

After assembly, clean reads were mapped to unigenes using Bowtie2 (v2.2.5) [27], and then gene expression level was calculated with RSEM (v1.1.12) [28]. To compare the difference of gene expression among different samples, the FPKM (Fragments per kilobase per transcript per million mapped reads) method was used [29]. The DEseq2 (Fold Change > = 2.00 and Adjusted Pvalue <= 0.05) and PossionDis (Fold Change > = 2.00 and FDR < = 0.001) were proposed to identify DEGs, and the *P*-value and FDR (false discovery rate) for each gene were calculated. DEGs were required to have thresholds of “log2 ratio ≥ 1” and “FDR < 0.001” [30]. Next, GO and KEGG analysis were again performed on the DEGs.

### Phylogenetic analysis

The phylogeny was inferred using the Neighbor-Joining method [31]. The bootstrap consensus tree inferred from 500 replicates is taken to represent the evolutionary relationship of the taxa analyzed [32]. Branches corresponding to partitions reproduced in less than 50% bootstrap replicates are collapsed. The percentage of replicate trees in which the associated taxa clustered together in the bootstrap test (500 replicates) are shown next to the branches. The analysis involved 38 terpene synthase and 52 CYP450 nucleotide sequences, respectively. All positions containing gaps and missing data were eliminated. There was a total of 479 and 1094 positions in the final dataset, respectively. Evolutionary analyses were conducted in MEGA7 [33].

### qRT-PCR analysis

Total RNA was extracted as indicated above. Each RNA sample was treated with RNase-free DNase (Tiangen, Beijing, China) following the manufacturer’s protocol in an effort to remove any residual genomic DNA (gDNA). DNase-treated RNA (2 mg) was subjected to reverse transcriptase reactions using M-MLV reverse transcriptase (Tiangen, Beijing, China) according to the manufacturer’s instructions. The sequences of the specific primer sets are listed in Additional file 2. The Actin (CL5382.Contig2_All) gene was used as an endogenous control, qRT-PCR was performed in 96-well plates in a Bio-Rad CFX96 real-time PCR system (Bio-Rad, CA, USA) with a SYBR Green-based PCR assay. The final volume for each reaction was 20 μL with the following components: 2 μL diluted cDNA template (1 mg/mL), 10 μL SYBR Green Mix (Bio-Rad, CA, USA), 0.4 μL forward primer (10 μM), 0.4 μL reverse primer (10 μM) and 7.2 μL ddH_2_O. The reaction was conducted under the following conditions: 95 °C for 3 min, followed by 40 cycles of denaturation at 95 °C for 10 s and 60 °C for 30 s. The melting curve was obtained by heating the amplicon from 65 °C to 95 °C at increments of 0.5 °C per 5 s. Each qRT-PCR analysis was performed with three biological replicates. The relative quantification of gene expression was computed using the 2^-ΔΔCt^ method.

## Results

### Active ingredient content in different tissues of *C. angulatus*

Prof. Wenjun Wu have detected the active ingredient content in different tissues of *C. angulatus*. He extracted the adhesive paste of the root bark, bark, xylem, leaves and fruits of *C. angulatus* and determined the bioactivities of the extracts by feed poisoning method. As a result, the active ingredients of *C. angulatus* were mainly distributed in the root bark, secondly in the leaves. However, bark and xylem contained little active ingredients, and the extracts from the fruit stimulated the growth of larvae of *Mythimna separate* at low concentrations (Table 1) [34] .

**Table 1:** Effects of extracts from different tissues of *C. angulatus* on larvae of *M. separate* [34]

Tissues	Extraction rate /%	Concentration in feed /(mg/kg)	Weight of larvae 7 days later /mg	Growth inhibition rate /%
Root bark	7.5	200	2.2	91.6
Leave	8.4	200	12.1	53.3
Xylem	1.6	200	18.1	30.0
Bark	3.7	200	19.5	24.6
Fruit	16	200	31.4	−21.5

### *C. angulatus* transcriptome sequencing and unigene assembly

In order to explore gene expression profile of *C. angulatus*, six cDNA libraries prepared from three leaf tissues (Leaf_1, Leaf_2 and Leaf_3) and three root tissues (Root_1, Root_2 and Root_3) were sequenced using the BGISEQ-500 platform. A total of 110.16, 109.15, 109.87, 109.56, 110.68, and 109.52 Mb clean reads were obtained after eliminated the low-quality reads (Table 2). Subsequently, the assembly of those clean reads were carried out and 77,255, 67,225, 69,274, 60,493, 56,701 and 60,373 unigenes were acquired by Trinity with a mean length of 970, 961, 961, 927, 839 and 949 bp respectively (Table 3).

**Table 2:** Summary of data output quality of various libraries

Sample	Total Raw Reads (Mb)	Total Clean Reads (Mb)	Q20%	Q30%
Leaf_1	135.45	110.16	95.78	87.48
Leaf_2	129.99	109.15	96.09	88.03
Leaf_3	132.88	109.87	95.98	87.93
Root_1	135.37	109.56	95.94	87.91
Root_2	135.41	110.68	95.97	88.10
Root_3	129.81	109.52	96.33	88.83

Q20: The percentage of bases with a Phred value > 20

Q30: The percentage of bases with a Phred value > 30

**Table 3:** Summary of assembly results of *C. angulatus*

Sample	Total number	Total length	Mean length	N50	GC(%)
Leaf_1-Conting	109,851	94,892,188	863	1648	41.71
Leaf_2-Conting	95,389	82,261,657	862	1593	41.82
Leaf_3-Conting	98,035	84,748,426	864	1609	41.67
Root_1-Conting	86,993	72,550,947	833	1538	42.16
Root_2-Conting	83,316	66,867,324	802	1460	42.41
Root_3-Conting	86,799	73,974,071	852	1589	42.27
Leaf_1-Unigene	77,255	74,938,903	970	1720	41.65
Leaf_2-Unigene	67,225	64,632,431	961	1657	41.76
Leaf_3-Unigene	69,274	66,633,515	961	1659	41.62
Root_1-Unigene	60,493	56,082,948	927	1583	42.11
Root_2-Unigene	56,701	50,673,238	893	1504	42.38
Root_3-Unigene	60,373	57,304,625	949	1637	42.21
All- Unigene	104,950	125,979,466	1200	2011	41.61

N50: a weighted median statistic that 50% of the Total Length is contained in Transcripts great than or equal to this value

**Fig. 2:**
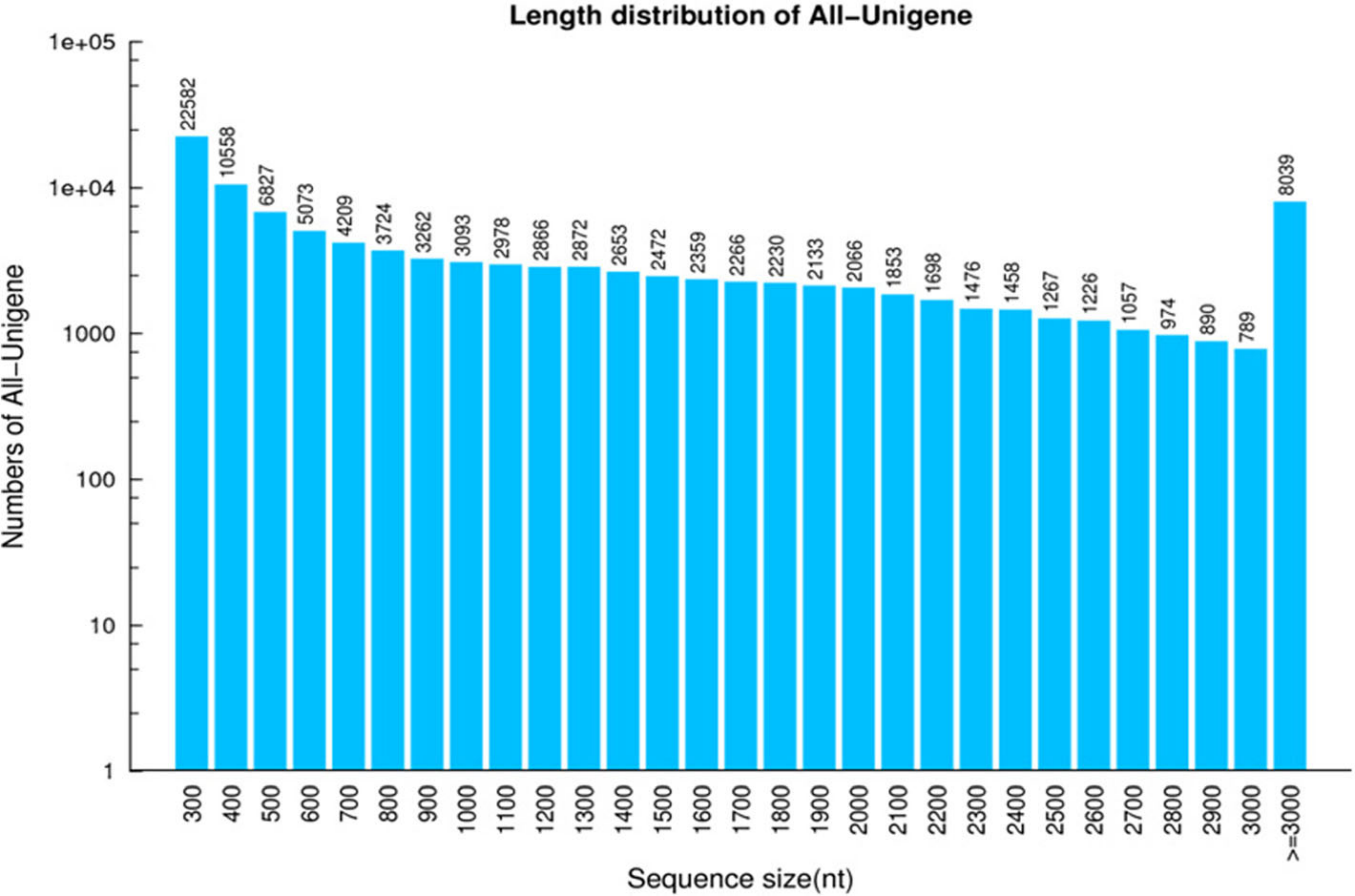
Distribution size of de novo assembled unigenes for *C. angulatus*. A total of 104,950 unigenes sizes were calculated for *C. angulatus*. This figure was generated with R 3.2.5

After de novo assembly of two *C. angulatus* tissues, 104,950 unigenes were finally obtained with an average length of 1200 bp (Table 3). Among these, 48,715 unigenes have a length longer than 1 kb (> 1000 bp) and 16,934 unigenes have a length range between 1000 bp and 1500 bp. A detailed summary of the sequencing and assembly results is shown in Table 3 and the length distribution of all unigenes is shown in Fig. 2.

### Functional annotation of *C. angulatus* unigenes

The contigs in six transcriptome sequencing databases were integrated and assembled into a total of 104,950 unigenes. After the assembly, annotations for the assembled unigenes were carried out via BLAST in seven public databases (NR, NT, Swissprot, KEGG, KOG, InterPro and GO), a total of 66,193, 53,315, 44,141, 48,810, 51,817, 56,377 and 39,866 were aligned, respectively. There were 71,479 unigenes (68.11% of all unigenes) annotated within at least one functional database. (Additional file 3).

### Functional classification of *C. angulatus* unigenes by KOG, GO and KEGG

**Fig. 3:**
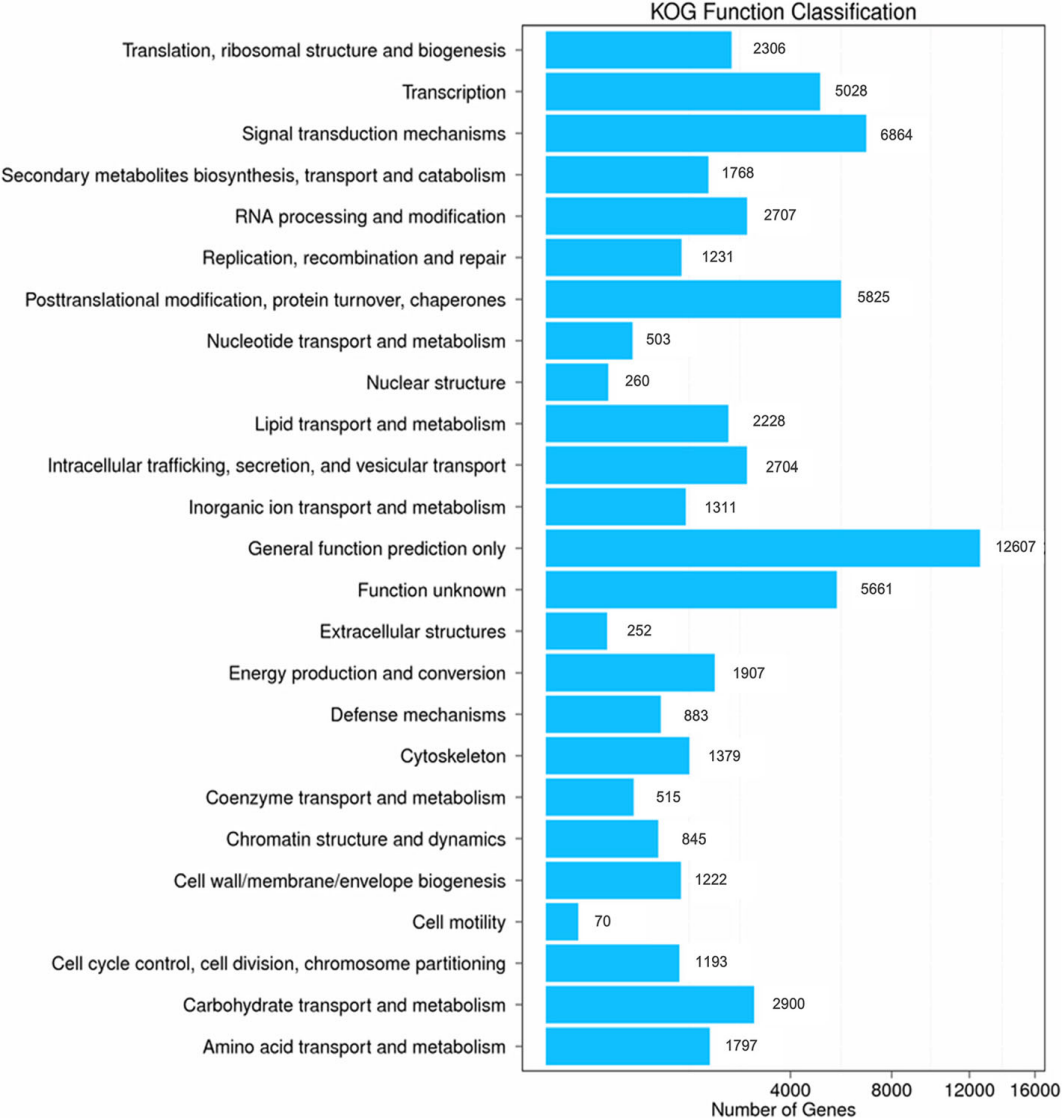
KOG function classification of *C. angulatus*. A total of 51,817 unigenes were classified into 25 functional categories according to their predicted gene products using the COG database. This figure was generated with R 3.2.5

KOG classification was used to further evaluate the completeness and effectiveness of the *C. angulatus* annotation. 51,817 unigenes were classified into 25 functional classifications (Fig. 3). The most dominant term was “General function prediction only” and 12,607 unigenes (24.3%) matched it, followed by “Signal transduction mechanisms” (6864, 13.3%), “Posttranslational modification, protein turnover, chaperones” and so on. Notably, 1768 unigenes in the “secondary metabolites biosynthesis transport and catabolism” category may play important roles in the biosynthesis of Celangulin V and other secondary metablites.

**Fig. 4:**
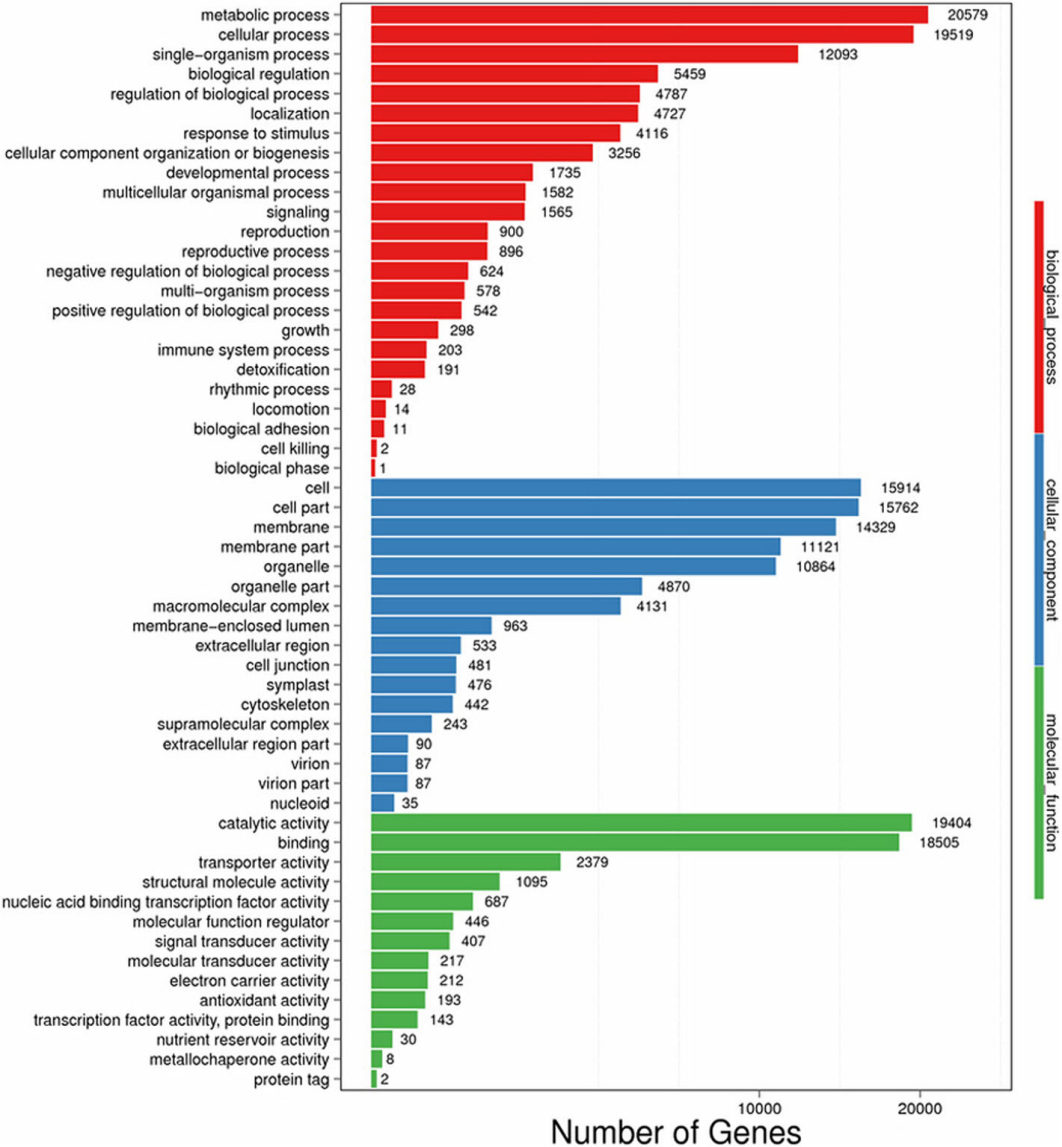
GO categories of *C. angulatus*. The results are summarized in mainly three categories: biological process, cellular component and molecular function. This figure was generated with R 3.2.5

GO analysis is an international standard system of gene function classification, and the mainly terms including “biological process”, “cellular component” and “molecular function”. Among the 104,950 unigenes, 39,866 unigenes were assigned to 55 GO terms. 24 groups were involved in biological process, in which “metabolic process” and “cellular process” occupied the mainly categories. 17 groups were mainly existed in “cellular component” and the high percentage of unigenes was associated with “cell”, “cell part” and “membrane”. 14 groups existing in “molecular function”, “catalytic activity” and “binding functions” play the most assignments in this category (Fig. 4).

A total of 48,810 unigenes were assigned to 135 KEGG pathways. The “metabolic pathways” represented the greatest group (9865 unigenes, 20.21%), followed by “biosynthesis of secondary metabolites” (5011 unigenes, 10.27%), including sesquiterpenoid and triterpenoid biosynthesis, terpenoid backbone biosynthesis, phenylpropanoid biosynthesis, cyanoamino acid metabolism, carotenoid biosynthesis and so on (Table 4). These pathways provide a valuable reference for investigating specific processes, functions and pathways during *C. angulatus* development.

**Table 4:** Pathways and number of unigenes related to secondary metabolites in *C. angulatus*

Biosynthesis of secondary metabolites pathway	Pathway ID	All genes with pathway annotation (48,810)
Anthocyanin biosynthesis	ko00942	29 (0.06%)
Benzoxazinoid biosynthesis	ko00402	30 (0.06%)
Betalain biosynthesis	ko00965	5 (0.01%)
Brassinosteroid biosynthesis	ko00905	54 (0.11%)
Caffeine metabolism	ko00232	16 (0.03%)
Carotenoid biosynthesis	ko00906	198 (0.41%)
Cyanoamino acid metabolism	ko00460	314 (0.64%)
Diterpenoid biosynthesis	ko00904	112 (0.23%)
Flavone and flavonol biosynthesis	ko00944	46 (0.09%)
Flavonoid biosynthesis	ko00941	231 (0.47%)
Indole alkaloid biosynthesis	ko00901	93 (0.19%)
Isoquinoline alkaloid biosynthesis	ko00950	126 (0.26%)
Monoterpenoid biosynthesis	ko00902	49 (0.1%)
Monobactam biosynthesis	ko00261	67 (0.14%)
Nicotinate and nicotinamide metabolism	ko00760	181 (0.37%)
Phenylpropanoid biosynthesis	ko00940	687 (1.41%)
Sesquiterpenoid and triterpenoid biosynthesis	ko00909	145 (0.3%)
Steroid biosynthesis	ko00100	225 (0.46%)
Stilbenoid, diarylheptanoid and gingerol biosynthesis	ko00945	175 (0.36%)
Terpenoid backbone biosynthesis	ko00900	310 (0.64%)
Tropane, piperidine and pyridine alkaloid biosynthesis	ko00960	142 (0.29%)

The sesquiterpene polyol ester Celangulin V is the major insecticidal active component in *C. angulatus*. 145 unigenes involved in sesquiterpenoid and triterpenoid biosynthesis were found in the KEGG pathways, and the detailed metabolic pathway is shown in Additional file 4. The pathway will be useful for further studies on the biosynthesis of Celangulin V.

### DEGs in the leaf vs root in *C. angulatus*

**Fig. 5:**
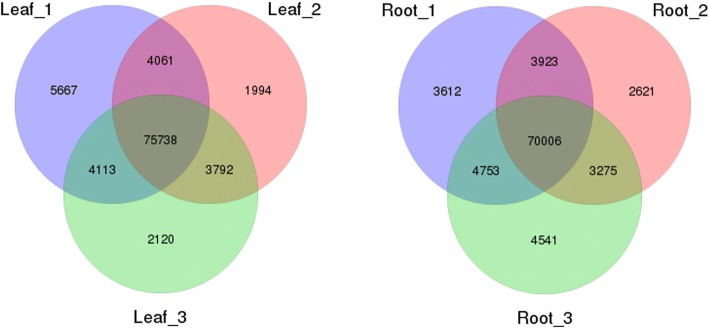
Venn diagram of the unigenes at Leaf and Root. This figure was generated with R 3.2.5

DEGs of the six transcriptome libraries were used to discover the unigenes with significant differences in expression. The expression of unigenes was calculated by FPKM. The different analysis methods were as follows: Leaf_1 Vs Root_1, Leaf_2 Vs Root_2, and Leaf_3 Vs Root_3 libraries were analyzed, and the DEGs were used to comment on all three replicates for GO classification and KEGG pathway analysis. A total of 19,440 DEGs were obtained, including 9024 up-regulated and 10,416 down-regulated unigenes in Leaf Vs Root. Furthermore, 5667, 1994 and 2120 unigenes expressed uniquely in Leaf_1, Leaf_2, and Leaf_3, respectively, and 75,738 unigenes were expressed in all three libraries, but at different levels; 3612, 2621 and 4541 unigenes expressed uniquely in Root_1, Root_2 and Root_3, respectively, and 70,006 unigenes were expressed in all three libraries, but at different levels (Fig. 5).

A total of 11,948 DEGs could be annotated in KEGG based on sequence homologies, which were annotated into 135 pathways and most genes were related to metabolic pathways (2854 DEGs) and biosynthesis of secondary metabolites (1764 DEGs). For the GO classification analysis, about 6366 7082 and 7890 unigenes could be annotated in “biological process”, “cellular component”, “molecular function” of GO terms respectively. Furthermore, the number of up-regulated genes was more than the number of down-regulated genes in the three pathways.

### Biosynthetic genes of terpenoid backbone in *C. angulatus*

Based on the KEGG annotation, a total of 310 contigs/unigenes are correlated with the terpene backbone biosynthesis, accounting for 0.64% among all genes with pathway annotation (48,810). Among them, 119 DEGs were discovered, accounting for 1% DGEs with pathway annotation (11,948). The unigenes encoding key enzymes involved in terpenoid backbone biosynthesis were listed in Table 5. 22 up-regulated unigenes among 26 DEGs were related to Mevalonate (MVA) pathway, including seven contigs/unigenes for acetyl-CoA AACT, two contigs/unigenes for HMGS, six contigs/unigenes for HMGR, two contigs/unigenes for MVK, two contigs/unigenes for PMVK, and three contigs/unigenes for MVD. 38 up-regulated unigenes among 49 DEGs were related to methylerythritol phosphate (MEP) pathway or 1-deoxy-D-xylulose-5-phosphate (DXP) pathway, including five contigs/unigenes for DXPS, three contigs/unigenes for DXPR, four contigs/unigenes for 4-hydroxy-3-methylbut-2-(E)-enyl-diphosphate synthase (ispG), three contigs/unigenes for IDI, 12 contigs/unigenes for isoprene synthase, and 11 contigs/unigenes for FPPS. These contigs/unigenes might participate in the biosynthesis of IPP that is the building block of terpenoids (Table 5).

**Table 5:** Discovery and expression of unigenes involved in celangulin V biosynthesis in *C. angulatus*

Enzymes name	Abbreviation	EC number	Expressed higher
Acetyl-CoA C-acetyltransferase	AACT	EC:2.3.1.9	CL9396.Contig2_All, Unigene26040_All, Unigene13706_All, CL270.Contig9_All, CL270.Contig8_All, CL270.Contig11_All, Unigene1229_All
Hydroxymethylglutaryl-CoA synthase,	HMGS	EC:2.3.3.10	CL1173.Contig1_All, CL1173.Contig2_All
Hydroxymethylglutaryl-CoA reductase	HMGR	EC:1.1.1.34	CL3155.Contig6_All, CL7171.Contig2_All, Unigene9669_All, Unigene28303_All, CL3155.Contig5_All, CL3155.Contig2_All
Mevalonate kinase	MVK	EC:2.7.1.36	CL11783.Contig1_All, CL11783.Contig2_All
Phosphomevalonate kinase	PMVK	EC:2.7.4.2	CL8165.Contig1_All, Unigene10676_All
Diphosphomevalonate decarboxylase	_	EC:4.1.1.33	CL9292.Contig1_All, Unigene24911_All, Unigene27813_All
1-deoxy-D-xylulose-5-phosphate synthase	DXPS	EC:2.2.1.7	CL4067.Contig9_All, CL4067.Contig8_All, CL4067.Contig7_All, CL4067.Contig5_All, Unigene2397_All
1-deoxy-D-xylulose-5-phosphate reductoisomerase	DXPR	EC:1.1.1.267	CL10481.Contig1_All, Unigene29109_All, CL10481.Contig2_All
4-hydroxy-3-methylbut-2-(E)-enyl-diphosphate synthase	ispG	EC:1.17.7.1 1.17.7.3	Unigene27344_All, CL2211.Contig12_All, CL2211.Contig16_All, CL2211.Contig17_All
4-hydroxy-3-methylbut-2-en-1-yl diphosphate reductase	ispH	EC:1.17.7.4	_
isopentenyl-diphosphate δ-isomerase	IDI	EC:5.3.3.2	CL674.Contig5_All, CL39.Contig4_All, CL674.Contig3_All
isoprene synthase	_	EC:4.2.3.27	CL4994.Contig7_All, CL4994.Contig2_All, CL4994.Contig6_All, CL4994.Contig8_All, CL4994.Contig9_All, CL686.Contig1_All, CL4994.Contig1_All, CL4994.Contig3_All, CL4994.Contig10_All, CL686.Contig2_All, CL4994.Contig4_All, Unigene2738_All
Farnesyl diphosphate synthase	FPP	EC:2.5.1.1 2.5.1.10	Unigene5517_All, Unigene20963_All, Unigene18861_All, Unigene28032_All, Unigene8091_All, Unigene43426_All, CL6922.Contig1_All, CL2794.Contig15_All, CL2794.Contig18_All, CL2794.Contig3_All, CL2794.Contig12_All
Germacrene D synthase Germacradienol synthase	_	EC:4.2.3.75 EC:4.2.3.22	CL7773.Contig1_All, CL8776.Contig2_All, CL8776.Contig1_All,
Valencene/7-epi-alpha-selinene synthase	_	EC:4.2.3.73 4.2.3.86	CL7773.Contig8_All
CYP71D9/CYP71D10/CYP71D11	_	EC:1.14.14.1 EC:1.14.14.19 1.14.14.32	CL8302.Contig2_All, CL12402.Contig1_All, Unigene922_All, CL5925.Contig2_All, CL12355.Contig1_All, CL12355.Contig4_All, CL5885.Contig1_All, CL5885.Contig2_All,
BAHD acyltransferase	_	EC:2.3.1.- EC:2.3.1.133	CL5679.Contig1_All, CL5679.Contig9_All Unigene21128_All, CL2580.Contig2_All

### Identification of sesquiterpene biosynthetic genes

**Fig. 6:**
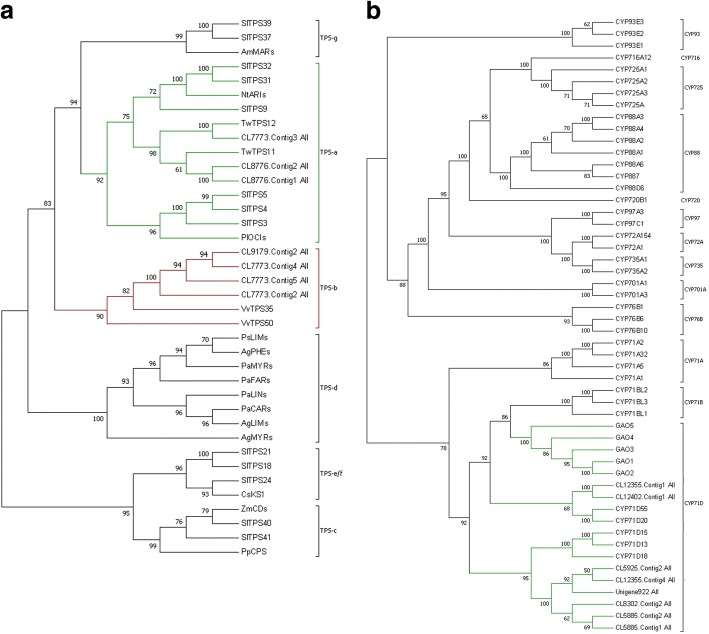
Phylogenetic analysis of terpene synthase, CYP450. **a** Phylogenetic tree of terpene synthase nucleotide sequence. **b** Phylogenetic tree of CYP450 nucleotide sequence. Accession numbers of proteins used in phylogenetic analysis are listed in Additional file 7. This figure was generated with MEGA 7.0.26

According to the KEGG annotation, there are 75 DEGs correlated with sesquiterpenoid and triterpenoid biosynthesis, accounting for 0.63% among 11,948 DEGs. 22 DEGs related to sesquiterpenoid biosynthesis were listed in Additional file 5, including 13 up-regulated and nine down-regulated unigenes, and involved metabolic pathways were shown in Additional file 4. These DEGs included genes encoding acyclic sesquiterpenoid synthase, bisabolene-type synthase, germacren-type synthase, humulene-type synthase and cadinyl-type synthase. Among all five types of sequiterpene synthases, the germacrene-type synthases have interested us. There are four types of germacrene-type synthases, including germacrene D synthases, germacradienol synthases, valencene synthases and 7-epi-alpha-selinene synthases. Totally, ten unigenes, including CL7773.Contig1_All, CL7773.Contig2_All, CL7773.Contig3_All, CL7773.Contig4_All, CL7773.Contig5_All, CL7773.Contig8_All, CL8776.Contig1_All, CL8776.Contig2_All, CL9179.Contig2_All, Unigene46663_All, encoding germacrene D synthases and germacradienol synthases expressed higher in roots and four unigenes, including CL12078.Contig1_All, Unigene4834_All, CL12078.Contig2, CL12078.Contig3_All, expressed higher in leaves. One unigene CL7773.Contig8_All encoding valencene synthase and 7-epi-alpha-selinene synthase expressed higher in roots and no unigenes expressed higher in leaves. CL7773.Contig8_All was annotated in all four germacrene-type synthases (Additional file 5). Besides, we performed phylogenetic analysis for the unigenes with representative plant sequiterpene synthases. The phylogenetic analysis revealed that CL7773.Contig1_All, CL8776.Contig2_All and CL8776.Contig1_All belonged to TPS-a subfamily, while CL9179.Contig2_All, CL7773.Contig4_All, CL7773.Contig2_All and CL7773.Contig5_All belonged to TPS-b subfamily (Fig. 6a).

In addition, in the downstream pathway of Celangulin V biosynthesis, some functional groups on the sesquiterpene skeleton are needed. Specifically, CYP450s and acyltransferases play major roles in this part. 288 unigenes were identified for the CYP450 family. Among them, 142 DEGs (88 up-regulated and 54 down-regulated unigenes) were discovered. There were 18 unigenes related to CYP 71 clan, including 13 up-regulated and five down-regulated unigenes. The 13 up-regulated unigenes encoding CYP71A1, CYP71A9, CYP71A25, CYP71D9, CYP71D10, CYP71D11, CYP71E7, CYP76A2, CYP83B1. The CYP71D9, CYP71D10, CYP71D11 have interested us, including CL8302.Contig2_All, CL12402.Contig1_All, Unigene922_All, CL5925.Contig2_All, CL12355.Contig1_All, CL12355.Contig4_All, CL5885.Contig1_All, CL5885.Contig2_All. (Additional file 6). A phylogenetic analysis of the eight unigenes with 44 plant cytochrome P450s involved in terpenoids biosynthesis including gibberellins, abscisic acid, carotenoids and plant defense substances. The phylogenetic analysis of CYPs revealed that all of the eight unigenes belonged to the CYP71D subfamily (Fig. 6b).

Two hundred thirty seven unigenes were related to acyltransferase family. Among them, 60 DEGs (33 up-regulated and 27 down-regulated unigenes) were discovered. There were four unigenes related to a plant acyl-CoA dependent acyltransferase superfamily, BAHD, including CL5679.Contig1_All, CL5679.Contig9_All, Unigene21128_All and CL2580.Contig2_All expressed higher in roots (Table 5).

### Validation and expression analysis of key genes

**Fig. 7:**
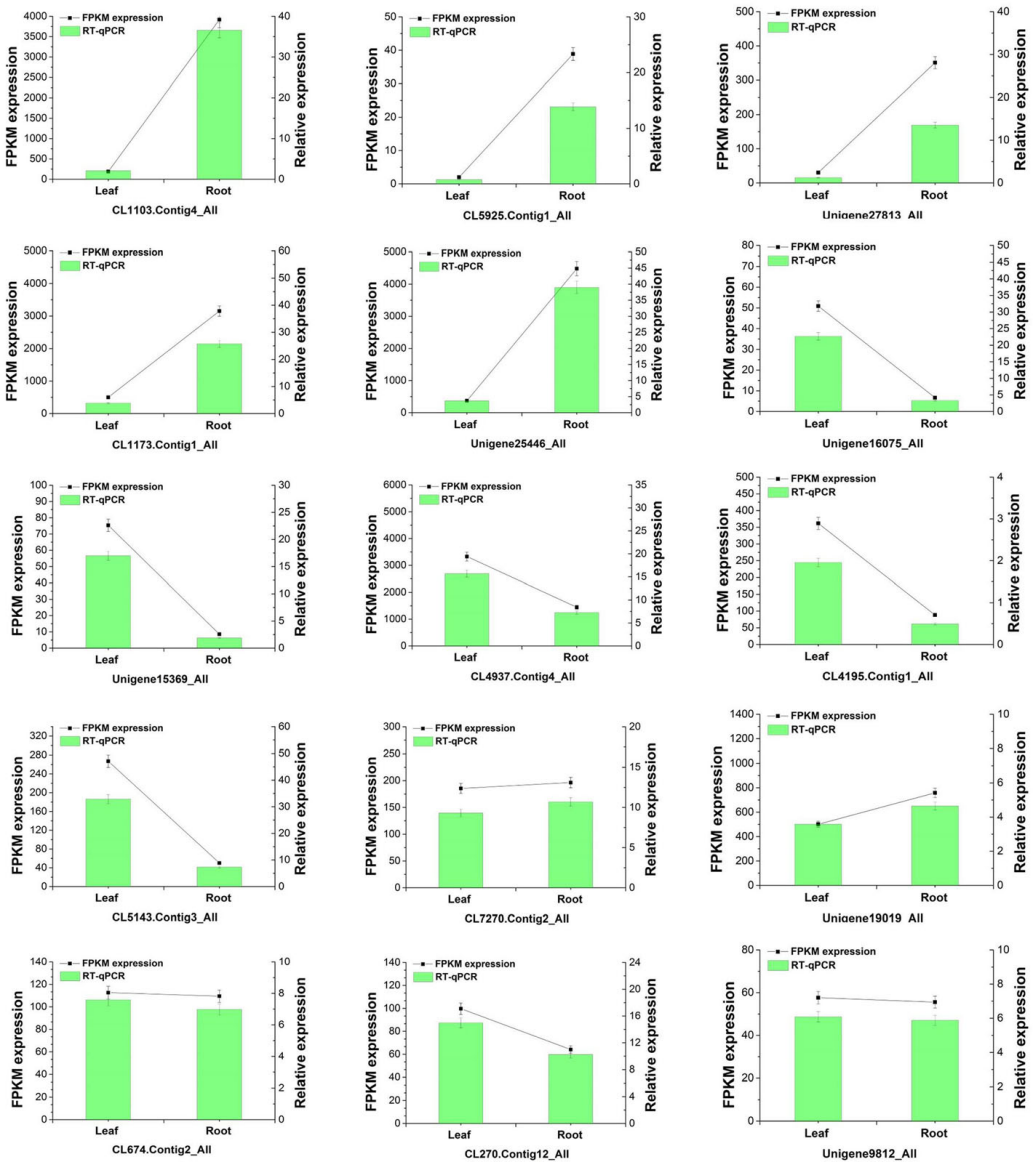
qRT-PCR validation of selected 15 DEGs at Leaf and Root. The relative expression level of each selected gene was determined by 2-ΔΔCT. Each bar represents the mean ± STD of triplicate assays. Values with different letters indicate significant differences at *P* < 0.05 according to Duncan’s multiple range tests. This figure was generated with OriginPro 8.5.1SR2

To confirm the accuracy of the BGISEQ-500 sequencing and FPKM calculated results, we selected 15 unigenes and used qRT-PCR to determine their relative expression level in the leaf and root tissues of *C. angulatus*. All 15 unigenes were putative sesquiterpene biosynthetic genes containing five higher-expressed unigenes (CL1103.Contig4_All, CL5925.Contig1_All, Unigene27813_All, CL1173.Contig1_All, and Unigene225446_All), five lower-expressed unigenes (Unigene16075_All, Unigene15369_All, CL4937.Contig4_All, CL4195.Contig1_All, and CL5143.Contig3_All) and five unchanged unigenes (CL7270.Contig2_All, Unigene19019_All, CL674.Contig2_All, CL270.Contig12_All, and Unigene9812_All) calculated by FPKM. The qRT-PCR and FPKM results were shown in Fig. 7, and the expression levels are similar.

## Discussion

### BGISEQ-500 sequencing and sequence annotation

*C. angulatus* is a kind of excellent traditional insecticidal plant due to their ample active ingredients of sesquiterpenes (β-agarofurans), alkaloids and flavonoids. Even though Celangulin V is considered as the most vital active constituents of *C. angulatus*, little is known about the biosynthetic and metabolic mechanisms of this sesquiterpene polyol ester. The aims of this study were to generate a large amount of cDNA sequence data that would facilitate more detailed studies on *C. angulatus*, and to identify the genes related to sesquiterpene polyol esters biosynthesis and metabolism. The availability of transcriptome data for *C. angulatus* will meet the initial information needs for functional studies of this species and its relatives. Six RNA-seqs were performed using BGISEQ-500 sequencing, which generated a total of 104,950 unigenes. 71,479 (68.11%) unigenes provided a significant BLAST result. This is the first report about transcriptome study of *C. angulatus*, providing adequate references to study on other plants with close relationship to *C. angulatus.*

### Terpenoid backbone biosynthetic genes and their differential expression patterns in *C. angulatus*

In previous studies on *C. angulatus*, Celangulin V were shown to be distributed in many organs, but mainly accumulated in root bark, the contents of Celangulin V showed higher in root than in leaf, and changed in different developmental stages [5–7]. We speculated that the genes expressed higher in root might encode some enzymes responsible for the biosynthesis of Celangulin V. Therefore, choosing the root and the leaf for comparative transcriptome analysis will greatly facilitate dissection of the genes involved in the organ-specific biosynthesis of Celangulin V. This approach is widely used for mining and identifying novel genes in biosynthesis of secondary metabolites in plants [35–37].

IPP and its isomer DMAPP, the universal biological precursors of all isoprenoids (basic C5 isoprene unit), can be obtained either through MVA/MEP pathways [38] (Fig. 1) in all eukaryotic cells and cytoplasm and mitochondria of plants or MEP pathway in bacteria, other prokaryotes and plastids in plants [39].

Totally, 22 up-regulated unigenes related to MVA pathway and 38 up-regulated unigenes related to MEP pathway possibly involved in terpenoid backbone biosynthesis are listed in Table 5. Numerous studies have been conducted with regard to engineer microorganisms for the production of different isoprenoids. The yeast *Saccharomyces cerevisiae* was always chosen as a microbial host to enhance the production of sesquiterpene by deregulating the MVA pathway, and the *Escherichia coli* was usually chosen as the microbial host for deregulating the MEP pathway. For example, the production of artemisinic acid engineered by MVA pathway achieved nearly 500 times production as previously reported [40]. Other natural products, such as patchoulol, farnesol, limonene and lycopene have also got higher titers through MEP or MVA metabolic pathway engineering methods [38, 41, 42]. In this study, the 22 up-regulated genes involved in MVA pathway and 38 up-regulated genes involved in MEP pathway were the candidate genes that can enhance the availability of the precursor (FPP) for biosynthesis of sesquiterpenes, and have the potential to improve the production of Celangulin V.

### Sesquiterpene biosynthetic genes and their differential expression patterns in *C. angulatus*

Terpenes, the largest class of natural products, are formed by terpene cyclases from linear oligoprenyl diphosphate precursors [43]. The phylogentic analyses of plant TPS protein sequences recognized seven major clades or subfamilies (TPS-a, TPS-b, TPS-c, TPS-d, TPS-e/f, TPS-g and TPS-h) [44, 45]. *C. angulatus* belongs to angiosperms. TPSs involved in angiosperm sesquiterpene synthesis are mainly distributed in TPS-a subfamily, with a small part dispersed in TPS-g/h subfamily. However, those involved in angiosperm monoterpene synthesis are mainly distributed in TPS-b subfamily [46]. CL7773.Contig1_All, CL8776.Contig2_All and CL8776.Contig1_All fell into TPS-a subfamily according to the phylogenetic analysis. Therefore, these unigenes maybe the sesquiterpene synthases and are predicted to be germacrene D synthase and germacradienol synthase [47, 48]. The CL7773.Contig8_All is predicted to be valencene/7-epi-alpha-selinene synthase, and the recombinant enzyme from *Vitis vinifera* gave 49.5% (+)-valencene and 35.5% (−)-7-epi-α-selinene [47]. These four unigenes maybe involved in the Celangulin V biosynthesis (Table 5).

Many studies have proposed the cyclization mechanisms of sesquiterpene synthases [49, 50]. The different cyclization processes can form diverse structures [51]. FPP cyclization reactions can proceed through C1-C6, C1-C7, C1-C10 and C1-C11 bond formation depending on which carbon-carbon double bond reacts with the initially formed allylic carbocation [52]. Celangulin V is a sesquiterpene polyol ester with β-dihydroagarofuran skeleton. Based on the chemical structural characteristics of β-dihydroagarofuran, we speculated that these four unigenes encoding sesquiterpene synthases could convert FPP to β-dihydroagarofuran through C1-C10 bond formation. In the future research, we will identify the function of candidate genes for sesquiterpene synthases belonging to the Celangulin V biosynthetic pathway by experiment.

### Celangulin V biosynthesis modified genes and their differential expression patterns in *C. angulatus*

Plant CYP450s [53–55] and acyltransferases [56] modify the sesquiterpene carbon ring and are important in biotechnology due to their ability to biosynthesize diverse secondary metabolites with various biological properties. The CYP71 clan is the largest P450 family and functionally diverse in all plant species [54], including a total of 110 members divided into 18 subfamilies and showing large clusters of duplicated genes and taxa-specific subfamily blooms [57]. The CYP71 clan is also called the cradle of monoterpenoid and sesquiterpenoid diversity. P450s involved in sesquiterpenoids metabolism have spawned exclusively in five families of the CYP71 clan: CYP71AV, CYP71BA, CYP71BL, CYP71D, CYP706B [58]. So far, the functions of many CYP450s have been identified, participating in the biosynthesis of terpenoid-based natural products in various plants [54]. At present, six bioactive sesquiterpenes have been successfully produced in heterologous systems by using CYP450 coding sequences [53]. In the capsidiol biosynthesis, 5-epi-aristolochene 1,3-dihydroxylase (CYP71D20) played a decisive role and the preferred reaction order of hydroxylation is at C1 followed by C3 [59]; CYP706B1 (part of the CYP71 clan) catalyzed hydroxylation of δ-cadinene at C8 in gossypol’s biosynthesis [60]; CYP71BA1 from *Zingiber zerumbet* (Zingiberaceae) catalyzed the conversion of a-humulene to 8-hydroxy-a-humulene at C8 in the zerumbone biosynthesis [61]; CYP71BL2 from *Lactuca sativa* [62] and CYP71BL3 from *Cichorium intybus* [63] specifically catalyzed 6a-hydroxylation by mediate hydroxylation of germacrene A acid at positions adjacent to the carboxy group. We obtained eight unigenes that were predicted to be the CYP71D9, CYP71D10, CYP71D11 may involve in Celangulin V biosynthesis (Table 5).

It is not well documented that acyltransferases are recruited as catalysts in the biosynthesis of plant specialized sesquiterpenoid compounds. Take artemisinin [40] and capsidiol [64] for examples, the acylated groups are not needed in their biosynthesis. However, some plant terpenoids need an acylation post-modification, which is depend on BAHD superfamily acyltransferases [65]. The BAHD acyltransferases are named after the first four biochemically characterized enzymes of the group, which use acyl-CoA thioesters as donor molecules [66]. For example, two BAHD alcohol acetyltransferases were identified from *L. x* intermedia glandular trichome database, which convert a variety of monoterpenes to monoterpene esters using coenzyme A as a cofactor [67]. Several taxoid-O-acetyl transferases (like TAX 9 and TAX 14), obtained from a previously isolated family of Taxus acyl/aroyl transferase cDNA clones, could catalyze some acylation steps of Taxol biosynthesis [68, 69]. We obtained four unigenes that were predicted to be the BAHD acyltransferase from transcriptome data, probably participating in biosynthesis of Celangulin V, according to acylated groups on C5, C6, C8, C9 and C14 positions of β-dihydroagarofuran skeleton.

Accordingly, we proposed the biosynthetic pathway of Celangulin V: β-dihydroagarofuran is the parent skeleton, highly oxygenated at C1, C3, C5, C6, C8, C9, C14 sites by one or more CYP450s to produce dihydroagarofuran polyols; Then, C5, C6 hydroxyl groups are acetylated by acetyltransferases; C9, C14 hydroxyl groups are butyrylated by butyryltransferase; C8 hydroxyl group is benzoylated by benzoyl transferase; C1, C3 are free. Based on our transcriptome sequencing, we annotated eight up-regulated CYP450 unigenes and four up-regulated BAHD acyltransferases in the sesquiterpene biosynthesis. In the future research, we will identify the function of candidate CYP450 and acyltransferase genes.

## Conclusions

Celangulin V is an efficient insecticidal sesquiterpenoid and widely used in pest control in China. In this study, leaf and root transcriptome were generated by next -generation sequencing, obtaining 104,950 unigenes with average length of 1200 bp. The transcriptome analysis revealed 16 unigenes probably involved in Celangulin V biosynthesis, in which four unigenes encoded sesquiterpene synthases (germacrene D synthase, germacradienol synthase, valencene synthase and 7-epi-alpha-selinene synthase), eight unigenes encoded CYP450s (CYP71D9, CYP71D10, CYP71D11) and four unigenes encoded BAHD acyltransferases. We further proposed the Celangulin V biosynthetic pathway. The transcriptome data not only provides valuable information on sesquiterpene polyol ester with β-dihydroagarofuran skeleton but also assists in mining genes involved in the production of other plants with close relationship to *C. angulatus*.

## Additional files

Additional file 1: The candidate transcript sequences that are involved in the biosynthesis of Celangulin V. A total of 16 unigenes were involved in the biosynthesis of Celangulin V, in which four unigenes encoded sesquiterpene synthases, eight unigenes encoded CYP450s and four unigenes encoded BAHD acyltransferases. (DOCX 22 kb)

Additional file 2: Genes and primers used for qRT-PCR analysis. Primers were designed using the Primer Premier program (version 5.0). (XLSX 22 kb)

Additional file 3: Summary of functional annotations of *C. angulatus.* The number of transcripts which be annotated with at least one functional database. (DOCX 15 kb)

Additional file 4: Putative Sesquiterpenoid and triterpenoid biosynthesis pathway of *C. angulatus*. Putative Sesquiterpenoid and triterpenoid biosynthesis of *C. angulatus* was constructed based on KEGG annotation. A total of 75 unigenes were involved in the metabolic pathway. These unigenes were distributed in the rectangular boxes in the figure. (DOCX 41 kb)

Additional file 5: 30 DEGs involved sesquiterpenoid biosynthesis in *C. angulatus.* The DEGs were assigned to KEGG biochemical pathways in sesquiterpenoid biosynthesis. (DOCX 17 kb)

Additional file 6: Summary of unigenes annotated as CYP450 and Acyltransferase. The details of CYP450 family, DEGs of CYP450 family, CYP71 Clan, Acyltransferase family, DEG of Acyltransferase family and BAHD Acyltransferases were showed in XLSX document. (XLSX 140 kb)

Additional file 7: Accession numbers of terpene synthase and CYP450 proteins used in the phylogenetic analysis. (DOCX 25 kb)

## Abbreviations

AACT: C-acetyltransferase
CMK: 4-(cytidine 5′-diphospho)-2-C-methyl-D-erythritol kinase
DEGs: Differentially expressed genes
DMAPP: Dimethylallyl diphosphate
DXP: 1-deoxy-D-xylulose-5-phosphate
DXPR: 1-deoxy-D-xylulose-5-phosphate reductoisomerase
DXPS: 1-deoxy-D-xylulose-5-phosphate synthase
FDR: False discovery rate
FPKM: Fragments per kilobase per transcript per million mapped reads
FPP: Farnesyl diphosphate
FPPS: Farnesyl diphosphate synthase
GO: Gene ontology
GPPS: Geranyl diphosphate synthase
HDR: (E)-4-hydroxy-3-methylbut-2-enyl diphosphatereductase
HDS: (E)-4-hydroxy-3-methylbut-2-enyl diphosphate synthase
HMGR: Hydroxymethylglutaryl-CoA reductase
HMGS: Hydroxymethylglutaryl-CoA synthase
IDI: Isopentenyl-diphosphate δ-isomerase
IPP: Isopentenyl diphosphate
ispG: 4-hydroxy-3-methylbut-2-(E)-enyl-diphosphate synthase
KEGG: Kyoto Encyclopedia of Genes and Genomes protein databases
KO: KEGG ortholog database
KOG: Clusters of euKaryotic Orthologous Group database
MCT: 2-C-methyl-D-erythritol 4-phosphate cytidylyltransferase
MDS: 2-C-methyl- D -erythritol 2,4-cyclodiphosphate synthase
MEP: 2-methylerythritol 4-phosphate pathways
MEP: Methylerythritol phosphate
MVA: Mevalonate
MVD: Mevalonate diphosphate decarboxylase
MVK: Mevalonate kinase
NR: NCBI non-redundant protein database
NT: NCBI non-redundant nucleotide sequence database
PMVK: Phosphomevalonate kinase
qRT-PCR: Quantitative Real-Time PCR
RIN: RNA Integrity Number
