# Supplementary material for: De novo leaf and root transcriptome analysis to explore biosynthetic pathway of Celangulin V in Celastrus angulatus maxim
Source: BMC Genomics. 2019 Jan 5;20:7. doi: 10.1186/s12864-018-5397-z (PMC6321707; doi:10.1186/s12864-018-5397-z)
Supplement: Supplementary file 1 — The candidate transcript sequences that are involved in the biosynthesis of Celangulin V. A total of 16 unigenes were involved in the biosynthesis of Celangulin V, in which four unigenes encoded sesquiterpene synthases, eight unigenes encoded CYP450s and four unigenes encoded BAHD acyltransferases. (DOCX 22 kb) [file 12864_2018_5397_MOESM1_ESM.docx]

**Additional file 1** The candidate transcript sequences that are involved in the biosynthesis of Celangulin V.

Sesquiterpene synthase:

CL7773.Contig1_All

TCATGTAATCTGGTAGTTGATCAATGGCACCAATGTCCCACCTCTCTACGGCATCGGTCAAGAGTCGGAGTTCTTCAATTGTACCATATGCATCGTATGTGTCGTCTAAAATTGATATCATTGTCACACTTTTAGTAAGAAAAATGCGGGTACGTCCATAATGTGGTTCAAAATTAGACCCAACAGTCCACATGTAGATTTCAGCAATTCTATCTCTTGCATAAGGAAATTTTGAAGCAATCTGCAAGTCTTTGTACCACCTTGAAACATGACTTAGCTCTTGTTGGTGCAACAATTGTACTCGATTAAAATCAATTTTCGCTAATTTGAGCATAATTTCATTTCGAGACTCATCTTCTTCGTAGAAATTGATGTATTTTCTCGACTCTATTCTTGGTATGCCCTTGTGATAAGGCTGTTGCAGTGCATGAGTAACTTGGTTTGCAAAGTGTGGGTTCAATTGGGTCACCATGGAATGAAGAGAAGTTTTTGAAAACACTAGTGCTTCATCCAAAACATCCTCTCCATGCATGCTCAAGTGAGAAGCTTCATACAAGCTTAGCATCCCCTCCACATCACTAGCTAGACTCATTTTAAACTTTCCATCATCTCCCTCAAACTTCTCAAACACGTCACAAGATATTTTATAACCATATTGTCTGAAAACGCGGAATAGAAGTGCTACAGCGTATAAACTGTAGTCATTATCATCGACGAGTTTAGTTTTGATTATGTTGAGATTCTGCATAAGCTGGTTTTCAATCTCACTTTCAAAGTGATATGAAATACCAAGACGACCCAACAAGTCAATCAACTCAATTTTCTCCACAATATCATTTGTAGACAACAACATATCATTCACTTGTACTTTCAACTCCTCCACTCTCTTTGTGTACGATCCGTACTCCGAGTCTTGATCAGAAGCAAAAGAAGCAAAGTCATCGTGAGCCCAAACGGCAGGGGCGAAGTTGGCCAACCGCCGTGGAGCTTCTACGGATTGGGTGGTTGCTGCCATTGAAATCAATATATGATTATGATATCAAGAAGAGTTTGTGTTTTAAGAAAAATGCCAAGGAAGTGCAATGCTGATATATCTGCTGAAGTGTGC

CL8776.Contig2_All

AAAGAATCAAGCCGATTGAAGATAATGAAATGAGAAAATTTCTTAGAGAAATTTTTGCTTAACCTTTTAAGACATTTAGTTGTCAAGAATTGTTCTTGATAAATGGTTTGATCATTTCTAGCTAGCACAAGAAATATCAAACACCCTTTCAATCCAATAATCCAGAGTTGATTTGCAACTTTGGCACTTCACACATCACTCCCAATCAAATTTCTCTATTTTATATCGTATATATGGCAACAAAAGCTGATCAGACAGTGCTCTCTTTACGTAGAACAGCGAATTTTCCTCCTGCAATATGGGGTCCTCAATACTTTGCTTCCATTTCCTTTGATGAATCGGTATTTGAATTGTACACCAAACAAGTGAAAGATTTGAAAATTCATGTTGAACATATGTTGGCCGATTCTACATTCAACATGGTGGAGAAAATCAAACTCATCGACTCGTTATGCCGTCTCGGTATATCATACCATTTTGAGAGTAAGATTGAAGATCAACTAAATCAAAATTTTGATTCCTTGCCTAAGTACCTGGTTGAAAATGATGTTTATGACCTAAACACGATTGCAATTTTATTTCGAGTATTCCGACAACATGGTTATAAAATGTCTTGCGATGTGTTTAACAAATTCAAGGATATTGATGGAAATTTCAAGGAGAGCTTAGTTAATGACGTCCATAGCATGCTAAGCCTGTACGAAGCTGCCCATTTGAGTACGCATATTGGAGAAGATAGTATTTTAGACGAATTCCTCACTTGCATGATTAGTCGCCTAAAATCTATGGAAAAGCAATGTGAACAACCTCATCTTGCAAAGCAAATAACTTATGCTTTGCAACAACCCCTTCACAAGGGCATACCAAGAATCGAATCGAGACAGTTCATCTCTTTTTATGAACAAGACGAGTCTCACAACGAGATGTTGATCAAGCTGGCTAAATTAGATTTTAACCGAGTACAATTATTGCACCAAAAAGAGCTTAGCCATCTTTCAAGGTGGTATGATAAGTTGGACTTTCCTTCAAATTTTCCTTATGCAAGAGAGAGAATTGCAGAAACTCACATTTGGTCTGTCGGAACTTATTTTGAGCATCAGTACTCTGATGCTCGAATAATTCTCACAAAAGTTATAACAATGATATCGATTCTAGATGACACATACGATCTTTATGGTACCATTGAAGAGCTCCGACTACTAACACTTGAAATACAGAGGTGGAACATAGATGCCATTGATAAGCTACCGGAGTACATGAAATGTCTCTATAATGTTCTTTTGAATGTTTATCACGAATTTGAGAATCAATTGGAAAGTGAAGGGAGATCTTATCTTGTCTCTTATGCAATAGATGCGATGAAAGAATTGTCGAGGGGCTACCTTGTTGAGGCGGAGTGGTTCCATGCAAGAATTGTGCCAACATTTGATGAATACTTGAAAAACGGATTAATCACAAGCAGTTATCACACAATCATATCAGCTTCTTTTCTAGGAATGGGAGAAATTGCGGGAATGGATGGTTTCGAATGGTTGAAAAGTAGTCCAAAGATTGTTACAGCTTCAATGGCAATCGGTCGTCTCATGAATGACCTAGTGTCGCACAAGGATGAGCAAAAAAAGGGGAGATGTTGCATCAGGAGTTGAGGCATATATGAAGCAACATGGCTCGTCTGAGACTGAAGCAATCAAGTATGTGGAGCAGAAGGCGGCGCATGCATGGAAAGATATCAATGAAGGATTTATGAGACAACTAAATAACATGTCCATGCAGCTTCTCATGCGAGGGCTCAACATTGCACGTGCGACTCATTTCTTTTACAAGGTTGATGATGCCTACACAAATTCAACTGCTTCAAACCACATTGTCGAGGCTTTGTTCATTGACCAGATTCCAATCCCAGAATAGAAGTCATTAAAGAACTTTGATTTTCAAGTTGGGCTTGTGATTGTAAAATAAGTGCTTGGAATGCTTTGATTTTAAGTTGGATTGATCAAGTAGGTGTGTGAGATGCATATATAGTGTAATGAATAATAGATGCACTACAAAGTAAATATTTTTTTTAGCGATGAAATTAGTGACAATATTATTAAGGATCAATTTGGGATGAAATTAGTGACAAAACTTTTCATCACTAAAATATTTCATCACTAATTCCG

CL8776.Contig1_All

AAAGAATCAAGCCGATTGAAGATAATGAAATGAGAAAATTTCTTAGAGAAATTTTTGCTTAACCTTTTAAGACATTTAGTTGTCAAGAATTGTTCTTGATAAATGGTTTGATCATTTCTAGCTAGCACAAGAAATATCAAACACCCTTTCAATCCAATAATCCAGAGTTGATTTGCAACTTTGGCACTTCACACATCACTCCCAATCAAATTTCTCTATTTTATATCGTATATATGGCAACAAAAGCTGATCAGACAGTGCTCTCTTTACGTAGAACAGCGAATTTTCCTCCTGCAATATGGGGTCCTCAATACTTTGCTTCCATTTCCTTTGATGAATCGGTATTTGAATTGTACACCAAACAAGTGAAAGATTTGAAAATTCATGTTGAACATATGTTGGCCGATTCTACATTCAACATGGTGGAGAAAATCAAACTCATCGACTCGTTATGCCGTCTCGGTATATCATACCATTTTGAGAATGTGTTTAACAAATTCAAGGATATTGATGGAAATTTCAAGGAGAGCTTAGTTAATGACGTCCATAGCATGCTAAGCCTGTACGAAGCTGCCCATTTGAGTACGCATATTGGAGAAGATAGTATTTTAGACGAATTCCTCACTTGCATGATTAGTCGCCTAAAATCTATGGAAAAGCAATGTGAACAACCTCATCTTGCAAAGCAAATAACTTATGCTTTGCAACAACCCCTTCACAAGGGCATACCAAGAATCGAATCGAGACAGTTCATCTCTTTTTATGAACAAGACGAGTCTCACAACGAGATGTTGATCAAGCTGGCTAAATTAGATTTTAACCGAGTACAATTATTGCACCAAAAAGAGCTTAGCCATCTTTCAAGGTGGTATGATAAGTTGGACTTTCCTTCAAATTTTCCTTATGCAAGAGAGAGAATTGCAGAAACTCACATTTGGTCTGTCGGAACTTATTTTGAGCATCAGTACTCTGATGCTCGAATAATTCTCACAAAAGTTATAACAATGATATCGATTCTAGATGACACATACGATCTTTATGGTACCATTGAAGAGCTCCGACTACTAACACTTGAAATACAGAGGTGGAACATAGATGCCATTGATAAGCTACCGGAGTACATGAAATGTCTCTATAATGTTCTTTTGAATGTTTATCACGAATTTGAGAATCAATTGGAAAGTGAAGGGAGATCTTATCTTGTCTCTTATGCAATAGATGCGATGAAAGAATTGTCGAGGGGCTACCTTGTTGAGGCGGAGTGGTTCCATGCAAGAATTGTGCCAACATTTGATGAATACTTGAAAAACGGATTAATCACAAGCAGTTATCACACAATCATATCAGCTTCTTTTCTAGGAATGGGAGAAATTGCGGGAATGGATGGTTTCGAATGGTTGAAAAGTAGTCCAAAGATTGTTACAGCTTCAATGGCAATCGGTCGTCTCATGAATGACCTAGTGTCGCACAAGGATGAGCAAAAAAAGGGGAGATGTTGCATCAGGAGTTGAGGCATATATGAAGCAACATGGCTCGTCTGAGACTGAAGCAATCAAGTATGTGGAGCAGAAGGCGGCGCATGCATGGAAAGATATCAATGAAGGATTTATGAGACAACTAAATAACATGTCCATGCAGCTTCTCATGCGAGGGCTCAACATTGCACGTGCGACTCATTTCTTTTACAAGGTTGATGATGCCTACACAAATTCAACTGCTTCAAACCACATTGTCGAGGCTTTGTTCATTGACCAGATTCCAATCCCAGAATAGAAGTCATTAAAGAACTTTGATTTTCAAGTTGGGCTTGTGATTGTAAAATAAGTGCTTGGAATGCTTTGATTTTAAGTTGGATTGATCAAGTAGGTGTGTGAGATGCATATATAGTGTAATGAATAATAGATGCACTACAAAGTAAATATTTTTTTTAGCGATGAAATTAGTGACAATATTATTAAGGATCAATTTGGGATGAAATTAGTGACAAAACTTTTCATCACTAAAATATTTCATCACTAATTCCG

>CL7773.Contig8_All

AGCTGCATGTTTTCTAGGCATGGGAGAAATTGCAGGAACAAAAGAATTTGAATGGCTCAAAAGCATCCCAAAAATTATTAGAGCTTCTGAGATGATCGGTCGTCTTATGGACGACATAATGTCACATAAGGAGGAGCAAGAGAGGGGGCATGTCGCGTCAAGTATTGAGTGCTTTATGAAGCAATATGGTGTGTCGAGTGAGGAAGAGGTGGTTCAAGATTTTCAAATTAGGATTGCGAATGCTTGGAAGGATATTAATGAAGAATGTGTGAG

CYP450:

CL8302.Contig2_All

GCTTCATTTTATGATTGATCCATCTATGCATTTAATTTTTATGACATTTCACTCTTTTGATACCAACCTTAATTATGCTGAAAATTGAAACATAACATTTTTATAAAGACCATATGTACATTATTCTAATTTTAAAGCTCATTAAGGCTTTAGGAGCGATAAGGAGTAGGAATTAAGTAGAGATCATCTTTTCTTCTCATCGATATGCCGAATAGATCCGTCATGGTAAGATCTTTTGGATTTGTCTCGTCGGGGAGTTTCCAATCAAAATGGTACAACAATTGTGAAAGCATGATGTCAACATTGGCAAGACCAAATGTCATGCCTGGACATATCCTCCTTCCGGCACCAAATGGAATGTACTCAAAATTAGTACCCTTGAACTCAATAGGATTATCTAGGAACCTCTCTGGGTAAAATCTTTCAGCATCAACCCAATACTCATCGTCTCTTCCGATCGCCCACACATTGACAAGTACTCGGGTTTTGGCAGGTATTTCATAGCCATCGATCACTTGTCGTTCTGCATTTTCTCTTGGAAGTAGAAGTGGAACCGGTGGGTGTAGTCTTAGAGTTTCTTTAATCACTAACTTCAAATACATCAACTCAGGAATCGCGGTTTCATCGAGACTTCCTCTATCGTTGAAAACCCGCCTTACCTCTGCTTGTGCCTTTTTCATCACTCTAGGGTCTTTAATCATTTCCACCATAGCCCAATCAACAGTTGCCGATGAAGTTTCACCTCCACCTCCAAAAACATCCTGAATTACGGCTTTGATATTGTTGACCGTTAATGGGTATTTAACATCCGGTTTGTGAAACTTGAGAAGAACATCAACCAAATCTTCAGCTTGTTCGCAAGCTATCCCGTCCTCAGGCGGTATTGCCTTGGCCTTAATGTGTTGGTTGATAATGTTTTGAAGAATGGTAGAGCTTCTTTGGTGAACATTTTCCAACCTAGAGCTAAACCAACTAATCAAATGAAGGAATTTCAAGGAAGGAAACACATCTCCTATATTGAATCCTCCTACTAGGTCTATAGATTCCTTAATAACCGATACGAGCTCATCCTTCTCTTCAGATTCGCCTCCCAACGCCACCCTTGAAACAATCGTATACGTCGATGAGAATACCTTTTCAGTCAAGTTGATTGGTGATCCTCCTGCTGCGTTCGAGACAACCCAATTGATCAACTTCGACATCTCTTGTTCTCTTATTGATCGAAACGATTGGATTCGTTTCATTGTGAAAAGCTCCATCAAGGAAATTTTCCGCAGCTGCCTCCAATACTCGCCATATGGTGAAAAAGTAATGCTAGTGAAATCATAAGTAAGGATTTTCGCTGCGTTTAACTTAGGCCTTGAAGCAAACACTTTGTCATGAGTTTTCAATACCTCTTTCGCGTACTTTGCCGAAGAAACAACAAGGGTAGGAACTTCACCAACTTGCAGGTACATCAACGGTCCATACTTCTTGGCCAAATCGCGTAGAGCAAGGTGGGGTTGAGAGCCAACCATTTGATGCAAGTTTCCTATAAGAGGTAACTTCCATGGCCCGGGAGGTCGTTTTGAAGTTGATTTAGATCTTTTGACTACTTTAAACACCATTAATGTAAAGAAAATGAAGCATGCAAGGATTAGGAAGTAGGAACAGTGGAGTTCCATGGAGCAAGTAGAGATTGTGATGAAGAAGGTTATGGTTGATAAGAAATGTGTGCAACTTTGCC

CL12402.Contig1_All

TGAGTATCCAAATTCATTTCAGTTTTCCTCATGGAGTTTGATTTTCTCTCCTCCTTGATCCTACTCAGTTTCATCTTCTTCATGTTTAAGATACTGAAAACATGGAAGATGTCGAATTCAAACAAGAATCTGCCTCCCGGGCCATGGAAGCTACCTTTGATTGGAAACTTGCACCAATTAGCTGGCTCTCAACCTCACCAAGCGCTACGAGACTTGGCAACGAAGTATGGACCGTTGATGTACTTACGACTAGGAGAAGTTCCTACTCTCATTGTTTCTTCTGCAGAATATGCTAAAGAAGTAATGAAAACTCACGACGTTGTCTTTGCTTCGAGGCCTCCATTGACCGCAGTCAAGCTTATGACCTATGATTTCACAGATATTGTTTTTGCACCATATGGTGAATACTGGAGACAACTACGGAAGATCTGCACAATGGAGCTATTAAGCATGAAACGAGTCCAGTCGTTGCGCTCCATAAGAGAGCAAGAGGTGTCAAATTTGGTCGAATGGATTTCCTCGAATGCAGGATCCCCGATAAACCTTACAGAAAAAGTATTCTCATTGTCACACATGATTCTTTCTAAGGCAGCATTTAAAAAAGATTATGGTGAGTTGCTGCAAAAATTCGTACTACTCATGAAAGAAATTGGACAATTGACATCAGGATTCGACGTGTCAGCGGTGTTTCCTTCCATGACATTTCTTCGCCTGATTAGTCGGACGAACACCAAAATTATGAAGATACATCAACAGGTGGATGGGATTCTTGAATACATTATCAATGAACAACTAAAGCACAAGGCGACATCGATGACTAGTAAGAGCGAAGAAGATCAAGATTTAGTAAATGTTCTTTTGAAATTTCATGAACGGGGTGACCTTGATGTTTCATTGACTATCGATAATATCAAAGCAATACTTTTGGACATTTTTTCTGGTGGCAGTGACACATCGAGCATGACGGTTGACTGGTCTATGGTAGAAATGTTGAGAAAGCCAAGAATAATGGAAAAGGCACAAGCAGAGGTGAGACAGGTTTTCGATAGCAGAGGAAGAGTTGATGAGGCAGGCATTGTAGAACTGAAATATTTGAAGCTAGTAATTAAAGAAACACTAAGATTACACCCTCCGGCGCCATTACTAATTCCAAGAGAAAATCAGGAGAGCTGTGAAATCAAGGGATATGAAATACCTAGCAAAACTAAAGTGATGGTGAATGCATGGGCTATTGGAAGAGATCCCAAGTATTGGACTGACCCTGAAAGATATTACCCGGAAAGGTTTCTCAATAGTACGATAGATTTCAAGGGGGCTAATTTTGAATACATTCCATTTGGTGCTGGAAGAAGGGTATGTCCAGGAATAATGTTTGGTATTGCTAATGTTGAGCTTGCGCTGTCACAATTGTTGTACTATTTTGACTGGAAACTTCCCAACGGAATGAAGCCTGAAGATCTCTCCATGACCGAAGACTTTGGCATAACAATTAGAAGAAGAGATGATCTCTACGTAATACCAACTCCTTATCACCCTTCACCTAGTCACTAAACCTTCAAAGTCGATGTTTGTTCTTACTTTATTTCATGGGGATTAATAAATAGTTTGTATCAGTTCACAT

Unigene922_All

AAAAAAAACAACATATTCACTAGCAAAATAACTATGGGAGATTAATGCACATTTATCTTTACTGCAGCTACACAAGCATGGAAACTAAAACAACAGCACAAACTAAAAGAAAACTTACAAGGTGTCAAAAGGTAACAATTCTGTGCTTACTCGACAAGGATAGCATTGTAAGGAATGGGGATTAAATACAAATTATTTTTTCTTCCAACTGCACCACCAAAATTCTCAGTCATGTCTAGTTCTTCTTGATTGATGTTATTAGGTAGCTTCCAGTCAAAATGGTAGAGTAACTTTGCAAGTATAAGCTCAACAGTCACTATTCCAGATGACATGCCAGGGCACATTCTCCTACCAGAACCAAAAGGGATAAACTCAAAGTTGTTCCCCTTGTAGTCTATCGAACTATTGACGAATCTCTCCGGATAGAACTTCTCTGCATCAATCCAACATTTAGGATCTCTTCCGATACTCCATGCATTAATGATGATTTGGGTTTTGAGCGATATGTCATATCCATTAATGTGGCATCTCTCTCTGCATTCTCTTGGGAGTAACAATGGAAGAGGAGGGTGTAACCTTAGACTTTCTTTGATAACTAATTTCAAGAATTCTAGTTCTTCTAGTCCGGCTTCATCTACATATCCTTTTCTGCCAAAGACCTGCCTCACCTCTGCTTGAGCCTTTGCCATCACTCTTGGATTCTTTAGCAATTCCGACATAGCCCACTCGGAAGCTGTAGATGTTGTTTCACTTCCAGCCATGAAAACTTCCATTACAAGTGCTTTGATGTTGTCTGCTGTTAAGGGGAACTGAAATTCTCCATGTGCCTGAAGCTTCAAGAGAACGTCTAAGAAATCATCAGTAAGCTCTACAGTGGAGTCGATTCTCGACTTGTTAACTCTATGTTCATGGATGAGGCTCTCGAGTATCTTATCGATGTCACGATGCAGCTTCTCAAGGCGAGTTCTCATCCCACTGACCAGATGAAACAACTTGACAGAAGGGAACATATCAGCCAAGCAGAAACCAGAGGTAACCTCTACAATTTTTCTTATAAGTGGTACAAATGATCCATGCTTATCACAACTCCCACCAAATGCTGTCTTTGTAGTAATTTCATATGTTAGACAAATTAACATCTCGCTGAGGTTGATCTTTGACCTTGCATTTGAAGAAATGGATTTAACAAATTGCGACACCTCTTCCTCTCTGATCGATCGCAAAGCTTGAACCCTTTACCGCTTAAGAGCTCCACTGTCAAAATTTTACGCATCTGTCTCCAGTAATCGCCATATGGTGAAAACCCCATATTACTAATGTTATAGAACAAGACCTCCATGGCAAGAAGATAAGGTCTGGATGCAAAAGTAATGTCATGGGTTTTCATTACTTCCTTGGCGATTTCTGGAGAAGAAACAACAATAGTGGATAATTCACCGAGCTGAAGATGCATAAGAGGTCCATATTTGTTGGATAAATCATGTAGGCGTCGATGCGGTAGAGAGCCAATCATTTGGTGAATGTTTCCTATCAGGGGTAGCTTCCATGGAGAAGGAGGAACAACTGAAACTGACTTATTGTCTTTTGATTTGCTCCATAGTCTCAGCAGCATCAATAAGAAGCCAAGGCAGGCCAAGATAATTGGAAAAGAAAGAATTTGATAATCCATGGTAAAGGTGG

CL5925.Contig2_All

GTTGGAATTAGTTGCATCACAAAAACATTCTAGTCCCTTGTCTTATAGACATTCATGATGTGCTGATAGGAAAAGGAGCTGGAAAAATAACATTTAGAATGGTTTGCTTGTAGTTTCACTCCACAATATCAGGATGATAAGGAATGGGAATCAAGTAGAGATTCATTTTTCTTCCAACTGCAGCACCAAATTTCTCAGTCATGTCCAGTTCTTCATATTTGACATTATCTGGGAGTTTCCAATCAAAATGGAACAACAGATTTGCGAGTGGAAGCTCGACGTTGGCGATTCCAAATGACATTCCTGGACACATCCTTCTTCCAGCACCAAATGGGATGTACTCAAAATTAGTCCCCTTGTAGTCTATTGAACTGTTGAGGAATCGTTCTGGATAGAACCTCTCAGCTTCAGTCCAATGATTCGGATCTCTTCCAATCGCCCATGCATTGATGACAACTCGGGATTTCGCCTGTATTTCATAGCCGTTTATATTACATTTCTCTCTACATTCTCTTGGAACTAACAATGGAACAGGTGGGTGTAATCTCATAGTTTCTTTGACAACCAACTTCAAGAATTTTAATTCCTCGAGACCTAGTTCATCGACATATCCTTTTGCACCAAAGACTTGCCTAACCTCTGCTTGTGCCTTTTCCATCACTTTCGGATTCCTCATCAGCTCTGATATTGCCCATTCCATAACTGTAGATGATGTCTCACTCCCACCCAGAATCATGTCCAGTATAACTGCTTTGACATTGTCGACTGTAATTTCAATCCCAAGATCTCCATTTTCTTGAAGATTCAAAAAAACATCTAGCAAATCGTCGTCCGTTGCATCTTCTCTAGAATCGGCCATTGCCTTCTTTAGTCTATGTTCTTTGATGATTTTTTGGAGGATCGCATCGGTCTTTTTATGCAGTATTTCAAGTTTAGTCCTCATGCCACTCATCGCTTGAAACAATTTAACAGAAGGGAAGAGATCTGCTAAGCTAAAACCAGCACCCAACTCTACACTTTCTTTTACCAGTGGTATAAATATTTCATGATCTTCAGTTCTCCTACCGAAGGCCGCTCTCGACGTGATCTCATACGTTAAAGCGATTAACATCTGACTAAGATTGATCTTTGATCCTGCCTTGGAATGAATTGAATTAACAAGCTCCTCTACCTCCTTTTTCCGAATTGGGATCATCGCTTGCACCCTTCTTGTGCTTAGCAGCTCCAGTGTACAAATTTTCCTCATTTGCCTCCAATACTCTCCATACGGTGCATATACAATGCTACAGGAATTGTATGAGAGGATCTGTGCTGCATTGAGAAAAGGCCTATCGGCGAAAGTGACGTCGTGGGTTTTCAATACTTCTTTGGCGACTTCTGGAGAAGAAACAATGATATTGGAGACTTCTCCAAGTTGAAGATGCATAAGAGGTCCATATTTCTTCGCCAAATCGCGCAGGCGGTGATGAGGTAGAGAGCAAATGAATTGGTGCATATTTCCTAGCAGAGGTAGCTTCCAAGGACCGGGAGGCTGTGGGCTTGCACTCGAGTTGTCTCTCTTTGATATTCTGAAAACCATAAACATGAAAGCAAAGAAGATCAGAAGTACTGGAAAAGAAGAAAATTGAAGATCCATGATGAGAAAGCAGGGGATGTTGGATGTTTTAAGTGTAGGAGTTTG

CL12355.Contig1_All

GCATCTTGCTTCTACAATCTCCATTTGCTCTACAGTCTGTTTAAAATCTTGATATCAACTGCAGCTTCATGGCCATCAAGGATCATCAAATATCTTCTTTTCCAGTTCTCTTGAGCTTTCTTGTTCTCATATTTATGCTCCTGAGAATATGGAATAAATCAAAAAACAAGAAGTCAAAGGTGGCAGCACCACCAGGTCCATGGAAGCTACCCCTTATAGGAAACATGCATCAAATGATTGGTTCTCTTCCCCATCGCCGCCTACGAGATTTAGCCTCAAAATATGGACCTATTATGCATCTTCAGGTAGGTGAAATCTCCACGATTATATTTTCTTCTCCAGAAGTTGCCAAAGAAGTAATGAAAACACATGACATTCATTTTGCCTCAAGACCTTATAGCCTTGTCGCGGATATCGTATTCTATAACTTTAAAGATGTTGGATTTGCGCCATATGGAGACTACTGGAGACAGATGCGTAAAATATGCACAGTGGAGGTATTCAGTGGAAGAAGGGTTCAAGCGGCACGACCAATTAGAGAAGAAGAGACATTGGAGTTCATTAAATCTATTTCTCTTAAGGCAAGGTCAAAAATTAACCTCGGCCAGATGTCAACTTGTTTGTCACTTGCAATCACATTGAAGGCAGCATTTGGTGAGAGTAGTGAGAGGTATGGAGCGTTTGTTCCACTTTTAAGTGAACTAATGGTGGTTATGGTAAATTTCAGATTACCTGATATGTTCCCATCCGTAAAATTATTTCAAAATATCATTGGCACGAGGACTCGACTTGAGAACCTGCATCATCGAATTGATCAGATACTTGAAAGCCTAATCAATGAACATAGACTTAACAGGTCAAGAATCAATTCGACTAATGCCGATAATTTCTTAGACATTCTCTTGAATCTTCAGGAAGACGGAGACTTCTCCTTTACAACAGACAACATCAAAGCAGTTGTGCTGGACATTTTCATTGGCGGGACTGAAACATCAGCTACAACTTTAGATTGGGCAATGTCAGAACTACTAAAGAATCCGAGAGTGATGGGAAAAGCTCAAGGAGAGGTGAGGCGGGTCTTTGGTAAAAAAGGATATGTAGATGAAGCAGGACTAGAAGAACTAAAATTCTTGACAGCAGTTATCAAAGAAACTCTAAGGTTACACCCTCCAGCTCCGTTGTTAATTCCAAGAGAATGTAGAGAGAGATGTCAGATTAATGGATATGAGATACCTGAAAAAACCCGCATTCTCATCAACGCATGGGTTATTGGAAGAGATCCCAACTATTGGACTGAAGCAGAGAGATTCTGTCCGGAAAGATTCCACGATAGCTCAATTGACTACAAGGGGAATAACTTTGAATATATCCCATTTGGTGCTGGAAGGAGGGCATGTCCTGGATTGTCATTTGGGATAGCTACTACTAAGCTTCCGCTTGCGCAGTTACTATGCCATTTTGATTGGAAACTCCCTAATAACATCAAACAAGAAGATCTAGATATGACTGAACGTCCTGGTATTACATTTGGAAGAAAAAGTGACTTAGTCTTGGTTCCCATTCCTTATAATTCCCCTGTTGTTAAGGAAAACGAGTGATGTTCACCTATGTTGCAATATTTTTTGTTTGTTACTCACAT

CL12355.Contig4_All

CTATTTTCATGGCCATGAAAATAGGGAAAAGATCCAAATCGACCTCAAAACTACCTCCCGGGCCATGGAAGCTACCTCTTATAGGAAACCTTCACCAAATGGTTGGCTCTCTACCCCACCATGCACTGCGCGATTTGGCGAAGAAGTACGGACCATTTATGTACCTGCAAATAGGACAAGTTCCCACTGTTGTTGTCTCTTCGTCAGATTACGCAAAAGAAGTAATGAAAACACACGACGTTAATTTTGCCAAAAGACCTTACAGCCTTGCCGCGGATATCATGTTCTATAATCTTAAAGATGTTCTATTTGCGACACATGGAGAGTACTGGAGACAGATGCGTAAAATATACACAGTGGAGGTCTTAAGTGGAAAAAGGGTTCAAGCGACTAGACCAATCAGAGAAGAAGAGACATTTGAGTTCATTAAATCCATTTCTCTCAAGGCAAGATCAAAAGTTAACCTCAGCCACATGTTAACTTCTTTAACATTTGCAATCACATTGAAGGCAGCATTTGGTGGGAGTAGTGAGAGACATGCAGCATTTGTTCCACTTTTAAGAGAACTAATAGAGGTTATCTCAGGTTTCAGTCTGGGTGATATGTTCCCAGACATAATATTCTTTCATATCATCAGTGGGATGAGGACTCGACTTGAGAGAATGCATCATGAAATCGATCAGATACTTGAAAGCATAATCAACGAACATAGACTTCACAGATCAAGAATCGATTCTGGTGATGCCGATAATATTTTAGACATTCTATTGAATCTTCAGGAAGATGGAGACTTCTCCTTCACAATAGACAACATCAAAGCAGTTATACTGGACATTTTCTTGGCCGGGACTGATGCATCCGCTACAACTTCAGAATGGGCAATGTCTGAACTGCTAAAGAATCCAAGAGTGATGGTTAAGGCTCAAGCAGAGGTGAGGCGGGTCTTTGATAAAAAAGGATATGTAGATGAGGCAGCACTTGAAAAACTAGAATTCTTGGAAGCAGTTATCAAAGAAACTCTAAGGTTACACCCTCCAGCTCCATTGTTAGCTCCAAGAGAATGTAGAGAGAGATGTCAGATTAATGGATATGAGATACCTGAAAAAACCCGCATTCTCATCAACGCATGGGTTATTGGAAGAGATCCCAACTATTGGACTGAAGCAGAGAGATTCTGTCCGGAAAGATTCCACGATAGCTCAATTGACTACAAGGGGAATAACTTTGAATATATCCCATTTGGTGCTGGAAGGAGGGCATGTCCTGGATTGTCATTTGGGATAGCTACTACTAAGCTTCCGCTTGCGCAGTTACTATGCCATTTTGATTGGAAACTCCCTAATAACATCAAACAAGAAGATCTAGATATGACTGAACGTCCTGGTATTACATTTGGAAGAAAAAGTGACTTAGTCTTGGTTCCCATTCCTTATAATTCCCCTGTTGTTAAGGAAAACGAGTGATGTTCACCTATCTCGCCATATTATTCAATGTGAGTAACAAACAAAAAATATTGCAACATAGGTGAACATCACTCGTTTTCCTTAACAACAGGGGAATTATAAGGAATGGGAACCAAGACTAAGTCACTTTTTCTTCCAAATGTAATACCAGGACGTTCAGTCATATCTAGATCTTCTTGTTTGATGTTATTAGGGAGTTTCCAATCAAAATGGCATAGTAACTGCGCAAGCGGAAGCTTAGTAGTAGCTATCCCAAATGACAATCCAGGACATGCCCTCCTTCCAGCACCAAATGGGATATATTCAAAGTTATTCCCCTTGTAGTCAATTGAGCTATCGTGGAATCTTTCCGGACAGAATCTCTCTGCTTCAGTCCAATAGTTGGGATCTCTTCCAATAACCCATGCGTTGATGAGAATGCGGGTTTTTTCAGGTATCTCATATCCATTAATCTGACATCTCTCTCTACATTCTCTTGGAGCTAACAATGGAGCTGGAGGGTGTAACCTTAGAGTTTCTTTGATAACTGCTTCCAAGAATTCTAGTTTTTCAAGTGCTGCCTCATCTACATATCCTTTTTTATCAAAGACCCGCCTCACCTCTGCTTGAGCCTTAACCATCACTCTTGGATTCTTTAGCAGTTCAGACATTGCCCATTCTGAAGTTGTAGCGGATGCATCAGTCCCGGCCAAGAAAATGTCCAGTATAACTGCTTTGATGTTGTCTATTGTGAAGGAGAAGTCTCCATCTTCCTGAAGATTCAATAGAATGTCTAAAATATTATCGGCATCACCAGAATCGATTCTTGATCTGTGAAGTCTATGTTCGTTGATTATGCTTTCAAGTATCTGATCGATTTCATGATGCATTCTCTCAAGTCGAGTCCTCATCCCACTGATGATATGAAAGAATATTATGTCTGGGAACATATCACCCAGACTGAAACCTGAGATAACCTCTATTAGTTCTCTTAAAAGTGGAACAAATGCTGCATGTCTCTCACTACTCCCACCAAATGCTGCCTTCAATGTGATTGCAAATGTTAAAGAAGTTAACATGTGGCTGAGGTTAACTTTTGATCTTGCCTTGAGAGAAATGGATTTAATGAACTCAAATGTCTCTTCTTCTCTGATTGGTCTAGTCGCTTGAACCCTTTTTCCACTTAAGACCTCCACTGTGTATATTTTACGCATCTGTCTCCAGTACTCTCCATGTGTCGCAAATAGAACATCTTTAAGATTATAGAACATGATATCCGCGGCAAGGCTGTAAGGTCTTTTGGCAAAATTAACGTCGTGTGTTTTCATTACTTCTTTTGCGTAATCTGACGAAGAGACAACAACAGTGGGAACTTGTCCTATTTGCAGGTACATAAATGGTCCGTACTTCTTCGCCAAATCGCGCAGTGCATGGTGGGGTAGAGAGCCAACCATTTGGTGAAGGTTTCCTATAAGAGGTAGCTTCCATGGCCCGGGAGGTAGTTTTGAGGTCGATTTGGATCTTTTCCCTATTTTCATGGCCATGAAAATAGAGAAGACGAAGCACAAAAGGATTGGGAATAATGAGGAAGATTGGAGTTCCATGGAGATGAGTTAGTAAAGATGAATTGTGAATTGGGATGAAGC

CL5885.Contig1_All

AACTAGTATGACATACACCCAAATATATAAACTACAAATATGTAACTACAATAAGTCCTCTGACGACGTATCCAAGTACTGCTGAAAACTAGGTAAAAGCTCATCCCTTACCTCCTACATATATATATTCGGTCAAGTGGCTGAGTTTGTTATTCAAAACATATTTTAGATACATAACTGAGATCATTTCAGTGAAGGGTAGTAAGGAGTTGGGATTAGGTAAAGGTCATCTTTTCTTCTAACCGTGACTGCAAATGATTCGGTCATGTCAAGATCTTCACCCTTCATTCCAGTAGGAAGTTTCCAATCAAAGTAGTACAACATTTGTGAAAGTGGAAGGTAAACATTTGCAAGACCAAATGCTATGCCAGGACATATTCTCCTTCCAGCACCAAACGGGGTGTATTCAAAATTAGTCCCCTTGTAGTCAATTGAATTATTGACGAATCTCTCTGGATAAAATGTTTCTGGATCAGTCCAATACTCGGGATCTCTTCCAATAGCCCAAGCATTCACAATAACTCTTGTTTTCGCTGGTATCGTGTAACCATTAATCTCACATTGCTCCAAACTTTCTCTTGGAACTAGTAAAGGCCCCGGAGGGTGTAGTCTTAGAGTTTCTTTGACGACCAATTTCAAATAGTTCAGTTCAGTAATGCCTGCTTCATCGACATTTCCTCTGGTATCGAAAACCTGTCTCACCTCGGCTTGTGCCTTCTTCAGTGTTCTTGGATTTTTGATCATTTCTGCAACAGCCCAATTAACAACCGTCGCGGATGTCTCACTTCCAGCACCAAACATGTCCTGAATTACTGATTTGATATTGTCGACAGTTAATGGAAACTCACCCCCTTCATGAAATTTCAAGAGAACATGGACTAGATCTTCCTCTGCGTCTAGTCTTCCAGTTTTTGAATCCATAATGTGTTGGTTGATAATTTTTTCAATAATCTCTTCACTTCGTTTATGTATCTTCTCGAATTTTGAGTTTACTCCAATAATCCAAGGAAGCCATTTCAAAGAAGGAAACAAATCTGAGACAATGAATCCTGATGCTAACTTTACGGCTTCCCTAACAAATGTTGCGAAGTCTTCTTGTCCACCATATGTCCGACCAAATGCAATCTTTGAAGTTAAGGTGTACGTTGATGAGTACAATTTTTCGGTCACGTTTATCGGTGATCCAGCATTTGAGCGAATCCATTCCATGAAATTCGACATCTCTTGCTCTCTTATTGGCCTATATGATTGGACACGTTTCGAGCTCAAAAGCTCTAGTGTGCAAATTTTCCGTAGTTGCCTCCAATATTCACCATATGGTGCAAACGCAATGTTAGTGTAATCATAAGTCAGGACTCTCATAGACTTTAGAGCTGGTCTCGAAGCAAAAACAAGGTCATGAGTTTTCATGACCTCTTTTGCATACTCTGCTGAAGAAGCGACCAATGTACCTGTCTCCCCAATTTGAAGGTGCATCAAGGGTCCATGCTTTTTGGCTAAGTCTCGTAGCGCTCGATGTGGTAGATCTTGTGCTAATTGATGTAGGTTTCCAATAAAAGGCAGCTTCCACGGCCCTGGAGGAAGATTTGAAGAACTTGATTTGGGTCTCATAACCATCTTCAGCACCACTAGAAAAAATAATAAAATGATGCTCGAAAGGATCGGGAAGGATGAGAGTTGGAGATCCATGAGGGAAAGGAAGCTTTTGATGGATTTGGAGCAAG

CL5885.Contig2_All

AAAATCTTCGCTCAACATTGACTAAACACATAATAATATTATTTATAACATATAACACAGGGAAACGTCTTCCATAACACTACCAAACAGACTTCCTTATTCCACATGCAATTGAGTTTCTAGACAAAACACTTGCCACTTCCTAATAATATTACATAAACACATGCGCGCGCGGCTGCGCTGAAAAATACAGACAAGAAATTTATATTATAGATAAACCAACAAGAACACAAGAGAGATATGTGGAGAAAATCCAGCATGGAACCTTGTGCTTGTTATCAACCGGAGAGCGAGCGATAAGGAGTTGGAACTAAGTACAGGTCATCTTTTGCTCTCATTGTTACATCGAAGCTTTCAATCATGTTGAGATCTTCAGGCTTCATTCCACCAGTGAAATTCCAGTCAAAATGGTACAACAGTTGTGACAGTGGAAACTCTACATTTGCAAGACCAAACGACATGCCAGGACACAACCTTCTTCCGGCACCAAATGGAGTATACTCAAAACTAGTCCCTTTGTAGTCAGCTGATTTATCGAGGAATCTCTCCGGATAAAAGCTTTCCGGTTCGGTCCAGTAGTTGGGATCTCTTCCAATAGCCCACGCGTTCACAATAACTTTGGTGTTGGCTGGTATCTCGTATCCATTGATCTCACATCGCTCCCAATTTTGTCTTGGAAGTAGGAAAGGCAACGGAGGATGAAGTCTTAGAGTTTCTTTAAGAACTAACTTCAAATATTTCAGCTCATGAACTCCTGTTTCGTCGACTCTTCCTCTGCTATCGAAAACTTGTCTCACCTCGGCTTGTACCTTTTTCATTACTCTTGGATTTTTCATCATTTCTCTCATAGCCCAATCGACAGTGGTCGCTGATGTTTCACCTCCAGCAACAAAAATGTCCTGAATTACCGATTTGATATTGTTAATACTCAATGGAAAGTCACCCTCCTCATGAAATTTCAAGAGAACATCAACCAGATCCTCATGTTCATCTTCATCTTCACCTTTTCCGGTTACTGGTTTCTTAACGTGTTGGTTTATAATGTTTCCAAGTAGTATGTCACCTTGTTTATGTAACCTTTCGGTTTTCCTGCCCTCGCCAATAATCCAATGAACCAATTTCAAGGAAGGAAACACATCCACGATATTGAATCCCGATGCTCTGTTTTGGGATGCCTTGGAATATTGGATGAATTCTTCATGTTCACCACGTCCACATGTCCTCCCAAATGCCACCTTTGAAACTAAGGCATATGTAGAAGAGTACAGTTTTTCCGTCAGGTTGATCGTTGATCCGGCATTCGAACCAATCCATTTGATTACATTCGACGTCTCTCGCTCTCTTATCGGCCTGAACGATTGAACACGTTTCACGCTCAAAAGCTCTATTGTGCAAATTTTCCTTAGTTGCCTCCAATACTCACCATATGGTGCAAAGGCAATGTCAGTGTGATCATAAGTCATGACTTTCATAGCATTTAAAAGAGGCCTCGAAGCGAAAGTAATGTCGTGAGTCTTCATCACCTCTTTTGCATAATCTGGTGAAGTAACAACAAGTGTTGGTACTTCCCCAAAATAAAGGTGCATCAAGGGTCCATGCTTTTTGGCTAAATTTTGTAGCGCTCGATGAGGTAGATCTTGTGCTAATTGGTGCAGGTTTCCGATTAAAGGTAGCTTCCATGGCCCAGGAGGCAGTTTTGAAGGCCTCAAAACTGACTTCAGCACCATTTGGATAAGTAAAATGATGCTCGAAAGGATCAGGAAGGACGAGAGTTGGAGATCCATGATGGAAAGAGTGTTGATGAATTTGGA

Acyltransferase:

CL2580.Contig2_All

TGGTGACTACTGAGGATTTTTTGTTGGCTGTTCAAGCCAATGTATTTGAATGTGGCGGAATGGCTATTGCAATATGCCTTACGCACAAGATTGTGGACGCAGGCTCTTTTTTTACATTCGTCAGAAAATGGGCTGCCATAGCTCGAGACTCAGACGACCGTGTGCTTCCGGACTTGACCATCGCGTCATCTCTTTTCCCTCCACGACGAGATTGTGTTTTGCCTACTTTCGAATTTCCTCGAGAAAAGTGCATGACAAGAAGGTTTGTTTTCAGTCCTTCGAGTCTCACGGCACTCAAAGCGAAGTGCACCTCTATAAACGTGCAACGCCCGACCAGAACAGAAGTGGTGGTGGCACTACTTTGGAAATGTCTAAT

CL5679.Contig1_All

CTCGAACACGTAGAGGTCACAAATACTACAAACACTAAAATGCCTTCTTCTTCTACCACTGTCATCTCCAAATGCACAATTCATCCAGACCAGAAATCCACCCTCAAGACATTGAAACTCTCTGTCTCTGACCTTCCTATGCTTTCCTGCCAGTACATCCAGAAGGGTGTTCTTCTTAATTACCCTCCTTACTCCTTTGAGGATCTCATTTTGTTCCTCAAGACCTCTCTCTCAGCATCCCTCTCTCACTTCCCGGCTCTCGCTGGCCGTCTCATAACAGACCAAGATGGCCATGTTCACATCTTATGTAATGATGCTGGAGTCGAATTCCTTGAAGCCAAGGCCAGACATTTAGACACGACCACGATTCTGTCACCAATTTATGTCCCTGACTGTTTCAAGGAGTTCTTCACTTTCGACAGGATGCTTAGCTATTCCGGCCACTCCAAGCCCTTGGTGGCTGTTCAAGTGACGGAATTAGCCGATGGTGTTTTCATCGGTTGCACTGTAAATCACGCGGTCACTGATGGAACCTCGTTTTGGCATTTCTTCAACACGTTCGCTGAAGTCTGTAAGGGAGCGAAGAAAATTTCGAATACGCCACAATTTAATCGCAACACAGTGTTTAACTCGCCGGCGGTGTTGGAATTCCCCGTTGGTGGACCAAAGGCGACAATCGCCTGCCACGAGCCGTTGCGAGAGAGGATCTTTCATTTTAGCAGAGAAGCGATCTTGAAGCTCAAATACAGAGCCAATAACGGTACTCTGTTACAGTGCGCAGAGATATTAGGGAAGCAATGTAACGACCGTTTGAAAGCCGTTAACGGAGAGGCAAACGGCAAGATAACTCCCGTTTCGGAAACTATACGAAAGAGCAATTGGACGTCGGAGATTTCTTCCTTTCAGTCTCTGAGCGCCCAGCTATGGCGCTCCGTGACACGTGCAAGAAAGCTCGCGCCGTCCAAAACGACCACGTTCCGAATGGCTGTAAACTGTCGACGCCGGCTCGAGCCGCAGCTCCACTCGTACTACTTCGGCAATGCGATACAGAGTATCCCCACCCTCGCTTCAGCGGGAGAGCTCCTGTCTCGCGATTTGTCCTGGGGAGCCGATCTTCTCCACAAAAACGTGGTGGCGCACGATGACGCGACCGTGCGACGCGGCATAAAAGATTGGGAGAGAGAACCGAGGGTTTTCCCGCTGGGGAATTTCGATGGGGCATCGATCACGATGGGAAGCTCGCCAAGATTTCCGATGTACGACAATGATTTTGGGTGGGGCCGGCCTGTTGCGGTGAGAAGTGGCAGGGCCAACAAGTTTGACGGTAAGATCTCAGCCTTCCCTGGGAGAGAAGGAAACGGAGCTGTCGATCTTGAAATTGTTTTGGCACCTTCTACGATGGCTGGGCTGGAGAGTGATCCAGAGTTCATGCAGTATGTATCAGCAATCAAATAATATATCTGGGCTTGTCATACAGGAACTTTAGTTGCATGGCGAAAGCCTCTGCATGTCCACTGTTGTGTTCCTCCACAAATCAAATTTCCGACTTGCTAAGCTTTATCTAG

CL5679.Contig9_All

AAAAAAACAAAATCATTTTCAAACATACCTTAAATATTGCAATAATTGGTCTCAATTCCCAATTGCTCAGTTAGAGACACCCGAATGTAAATAAATTCATAAGCTTCATATATCACGCATAATACATATGTAATTTTTTTGTACATATAACTCAACTAGATCTCTATTATAATAAGACTTTTGACTTTGAAAGTTATAAGCACTTTTGGTCAAAAGAGAGATTCAGACCAATCACAGGATTGCTGATACATACTGCATGAACTCTGGGTCACTCTCAAGGCCAGCCATCGTGGAAGGTGCCAAAACAACCTCAAGATCAACGGCTCCGTTTCCTTCTCTCCCAGGGAAGGCTGAGATCTTACCGTCAAACTTGTTGGCCCTACCACTTCTCACTGCAAGAGGCCGGCCCCACCCAAAATCATTGTCGAACATCGGAAATCTTGGCGAGCTTCCCATCGTGATCGATGCCCCATCGAAATTCCCCAGCGGGAAAACCCTCGGTTCTCTCTCCCAATCTTTTATGCCGCGTCGCACGGTCGCGTCATCGTGCGCCACCACGTTTTTGTGGAGAAGATCGGCTCCCCAGGACAAATCGCGAGACAGGAGCTCTCCCGCTGAAGCGAGGGTGGGGATACTCTGTATCGCATTGCCGAAGTAGTACGAGTGGAGCTGCGGCTCGAGCCGGCGTCGACAGTTTACAGCCATTCGGAACGTGGTCGTTTTGGACGGCGCGAGCTTTCTTGCACGTGTCACGGAGCGCCATAGCTGGGCGCTCAGAGACTGAAAGGAAGAAATCTCCGACGTCCAATTGCTCTTTCGTATAGTTTCCGAAACGGGAGTTATCTTGCCGTTTGCCTCTCCGTTAACGGCTTTCAAACGGTCGTTACATTGCTTCCCTAATATCTCTGCGCACTGTAACAGAGTACCGTTATTGGCTCTGTATTTCAACTTCAAGATCGCTTCTCTGCTAAAATGGAAAATCCTCTCGCGCAATGGCTCTTCACCGGAGAACGTCGCCTTTGGTCCACCGGCTGGGAATTCAAGCACTTCCGGCGAGTTAAACACTGTGTTGCGAGTAAATTCTGGAGTATTCGAAATTTTCTTCGCTCCCTTACAGACTTCAGCGAACGTGTTGAAGAAATGCCAAAACGAGGTTCCATCAGTGACCGCGTGATTTACAGTGCAACCGATGAAAACACCATCGGCTAATTCCGTCACTTGAACAGCCACCAAGGGCTTGGAGTGGCCGGAATAGCTAAGCATCCTGTCGAAAGTGAAGAACTCCTTGAAACAGTCAGGGACATAAATTGGTGACAGAATCGTGGTTGTGTCTAAATGTCTGGCCTTGGCTTCAAGGAATTCGACTCCAGCATCATTACATAAGATGTGAACATGGCCATCTTGGTCTGTTATGAGACGGCCAGCGAGAGCCGGGAAGTGAGAGAGGGATGCTGAGAGAGAGGTCTTGAGGAACAAAATGAGATCCTCAAAGGAGTAAGGAGGGTAATTAAGAAGAACACCCTTCTGGATGTACTGGCAGGAGAGCATAGGGAGGTCAGAGACAGAGAGTTTCAATGTCTTGAGGGTGGATTTCTGGTCTGGATGAATTGTGCATTTGGAGATGACAGTGGTAGAAGAAGAAGGCATTTTAGTGTTTGTAGTATTTGTGACCTCTACGTGTTCGAGGAAATGCCAGTGAGGAGCAAAGAGTATGGTTGGGTTTAAAT

Unigene21128_All

ATTTATGCGACGAAAACATATAGATTTGCTTGAAAACCCCTGCACTAACCTTGATGTGGTTATTGCACATTTCCAAAGAAGTGCTGTCACTGCTTCTGCTCTTGTGGGTTGTTGCACTCTTGTACTAGCGGTTTTAGCCTTCAGCTTTGCGATACTAGTAGAATTAAAAACAAACCTCTTCCTTGCACACTTGCCTCGACGACTAAAATCAAGCGGAGGCAATATTAACGAATTTTGTGGAGGAAGAAAATCTGATGGGACGAAAAATTCAGGATTATGCATTGCTGGAAATGACGACTCTCTAGCCGTGGAGAACCAACTCTTGACGAATGTGAAAAATGAGACTGCATCAGCTACCTTGTGGGAAA
