# Supplementary material for: De novo leaf and root transcriptome analysis to explore biosynthetic pathway of Celangulin V in Celastrus angulatus maxim
Source: BMC Genomics. 2019 Jan 5;20:7. doi: 10.1186/s12864-018-5397-z (PMC6321707; doi:10.1186/s12864-018-5397-z)
Supplement: Supplementary file 3 — Summary of functional annotations of C. angulatus. The number of transcripts which be annotated with at least one functional database. (DOCX 15 kb) [file 12864_2018_5397_MOESM3_ESM.docx]

**Additional file 3** Summary of functional annotations of *C. angulatus.*

| Values | Total | Nr | Nt | SwissProt | KEGG | KOG | InterPro | GO | Overall |
| --- | --- | --- | --- | --- | --- | --- | --- | --- | --- |
| Number | 104,950 | 66,193 | 53,315 | 44,141 | 48,810 | 51,817 | 56,377 | 39,866 | 71,479 |
| Percentage | 100% | 63.07% | 50.80% | 42.06% | 46.51% | 49.37% | 53.72% | 37.99% | 68.11% |

Overall: the number of transcripts which be annotated with at least one functional database.
