# Supplementary material for: De novo leaf and root transcriptome analysis to explore biosynthetic pathway of Celangulin V in Celastrus angulatus maxim
Source: BMC Genomics. 2019 Jan 5;20:7. doi: 10.1186/s12864-018-5397-z (PMC6321707; doi:10.1186/s12864-018-5397-z)
Supplement: Supplementary file 4 — Putative Sesquiterpenoid and triterpenoid biosynthesis pathway of C. angulatus. Putative Sesquiterpenoid and triterpenoid biosynthesis of C. angulatus was constructed based on KEGG annotation. A total of 75 unigenes were involved in the metabolic pathway. These unigenes were distributed in the rectangular boxes in the figure. (DOCX 41 kb) [file 12864_2018_5397_MOESM4_ESM.docx]

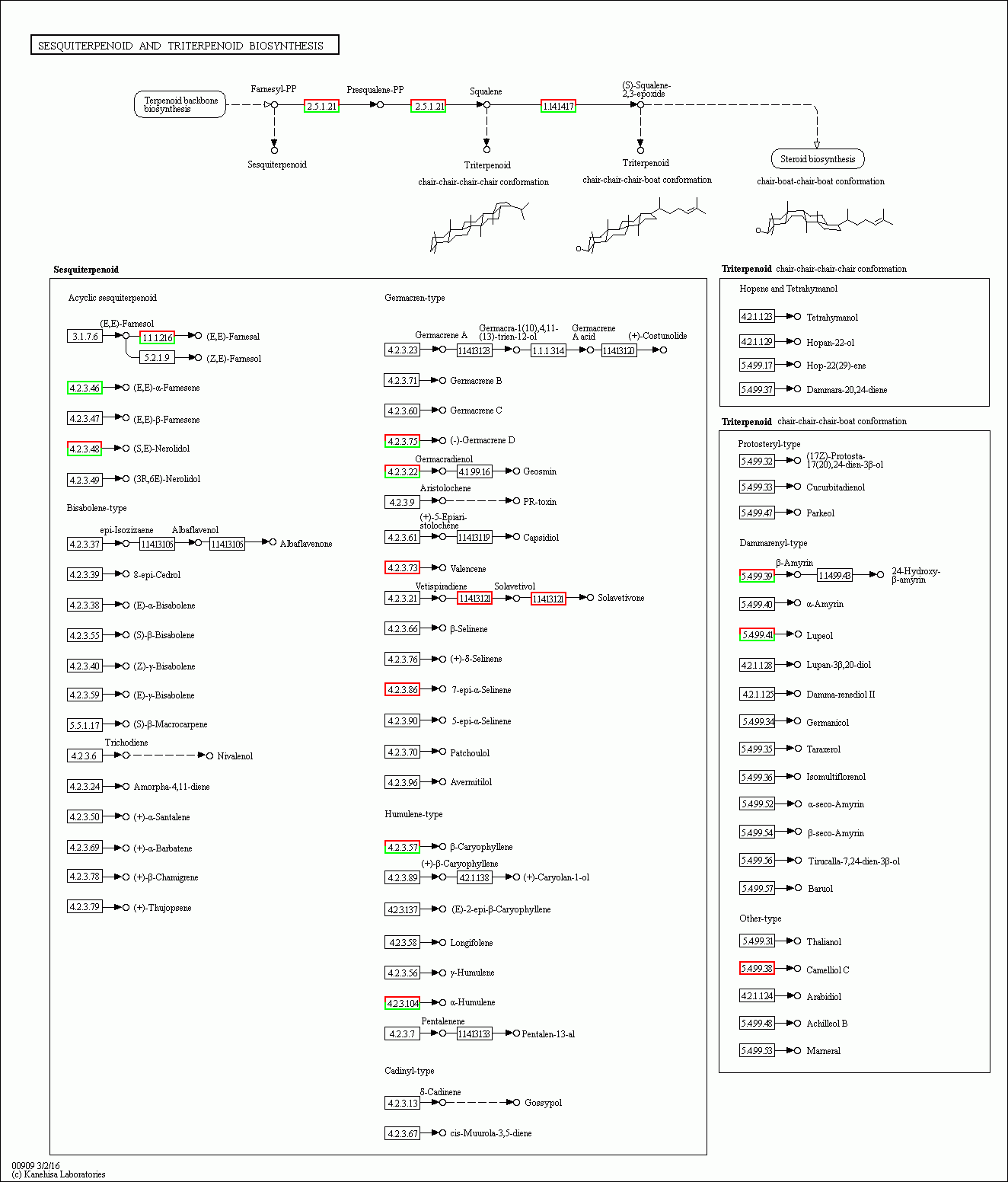


**Additional file 4.** Putative Sesquiterpenoid and triterpenoid biosynthesis pathway of *C. angulatus*. Putative Sesquiterpenoid and triterpenoid biosynthesis of *C. angulatus* was constructed based on KEGG annotation. A total of 75 unigenes were involved in the metabolic pathway. These unigenes were distributed in the rectangular boxes in the figure.
