# Supplementary material for: De novo leaf and root transcriptome analysis to explore biosynthetic pathway of Celangulin V in Celastrus angulatus maxim
Source: BMC Genomics. 2019 Jan 5;20:7. doi: 10.1186/s12864-018-5397-z (PMC6321707; doi:10.1186/s12864-018-5397-z)
Supplement: Supplementary file 5 — 30 DEGs involved sesquiterpenoid biosynthesis in C. angulatus. The DEGs were assigned to KEGG biochemical pathways in sesquiterpenoid biosynthesis. (DOCX 17 kb) [file 12864_2018_5397_MOESM5_ESM.docx]

**Additional file 5** 30 DEGs involved sesquiterpenoid biosynthesis in *C. angulatus.*

| Sesquiterpenoid-type | Enzymes name | Abbreviation | EC number | Expressed higher | Expressed lower |
| --- | --- | --- | --- | --- | --- |
| acyclic sesquiterpenoid | NAD+-dependent farnesol dehydrogenase | _ | EC:[1.1.1.354](http://www.kegg.jp/dbget-bin/www_bget?ec:1.1.1.354) | Unigene15856_All | CL854.Contig4_All,  CL4195.Contig1_All, |
|  | alpha-farnesene synthase | _ | EC:[4.2.3.46](http://www.kegg.jp/dbget-bin/www_bget?ec:4.2.3.46) | _ | Unigene20217_All  Unigene16075_All |
|  | (3S,6E)-nerolidol synthase | _ | EC:[4.2.3.48](http://www.kegg.jp/dbget-bin/www_bget?ec:4.2.3.48) | CL10218.Contig2_All | CL12179.Contig2_All |
| Bisabolene-type | _ | _ | _ | _ | _ |
| Germacren-type | Germacrene D synthase/ Germacradienol synthase | _ | EC:[4.2.3.75](http://www.genome.jp/dbget-bin/www_bget?ec:4.2.3.75) EC:[4.2.3.22](http://www.genome.jp/dbget-bin/www_bget?ec:4.2.3.22) | CL7773.Contig1_All,  CL8776.Contig2_All,  CL9179.Contig2_All,  CL7773.Contig4_All,  CL8776.Contig1_All,  CL7773.Contig3_All,  CL7773.Contig2_All,  CL7773.Contig8_All,  CL7773.Contig5_All,  Unigene46663_All | CL12078.Contig1_All, Unigene4834_All, CL12078.Contig2_All, CL12078.Contig3_All |
|  | Valencene/7-epi-alpha-selinene synthase | _ | EC:[4.2.3.73](http://www.genome.jp/dbget-bin/www_bget?ec:4.2.3.73) [4.2.3.86](http://www.genome.jp/dbget-bin/www_bget?ec:4.2.3.86) | CL7773.Contig8_All | _ |
|  | Premnaspirodiene oxygenase | _ | EC:[1.14.13.121](http://www.genome.jp/dbget-bin/www_bget?ec:1.14.13.121) | CL5925.Contig1_All | _ |
| Humulene-type | alpha-humulene/beta-caryophyllene synthase | _ | EC:[4.2.3.104](http://www.kegg.jp/dbget-bin/www_bget?ec:4.2.3.104)  [4.2.3.57](http://www.kegg.jp/dbget-bin/www_bget?ec:4.2.3.57) | CL9179.Contig2_All | Unigene4834_All,  CL12078.Contig2_All,  CL12078.Contig3_All |
| Cadinyl-type | _ | _ | _ | _ | _ |
