# Supplementary material for: De novo leaf and root transcriptome analysis to explore biosynthetic pathway of Celangulin V in Celastrus angulatus maxim
Source: BMC Genomics. 2019 Jan 5;20:7. doi: 10.1186/s12864-018-5397-z (PMC6321707; doi:10.1186/s12864-018-5397-z)
Supplement: Supplementary file 7 — Accession numbers of terpene synthase and CYP450 proteins used in the phylogenetic analysis. (DOCX 25 kb) [file 12864_2018_5397_MOESM7_ESM.docx]

**Additional file 7**

Accession numbers of terpene synthase proteins used in the phylogenetic analysis:

| Enzyme name | Accession No. | Species |
| --- | --- | --- |
| TPS-a  SITPS32  SITPS31  NtARIs  SITPS9  TwTPS12  TwTPS11  SITPS5  SITPS4  SITPS3  PIOCIs | JN412081  JN412082  AAA19216  JN408289  [KY193788.1](https://www.ncbi.nlm.nih.gov/nucleotide/KY193788.1?report=genbank&log$=nuclalign&blast_rank=1&RID=UYCCHS5X01R)  [KU948705.1](https://www.ncbi.nlm.nih.gov/nucleotide/KU948705.1?report=genbank&log$=nucltop&blast_rank=1&RID=UYCREZ3701R)  JN408286  JN408285  JN408284  ABY65110 | *Solanum lycopersicum*  *Solanum lycopersicum*  *Nicotiana tabacum*  *Solanum lycopersicum*  *Tripterygium wilfordii*  *Tripterygium wilfordii*  *Solanum lycopersicum*  *Solanum lycopersicum*  *Solanum lycopersicum*  *Phaseolus lunatus* |
| TPS-b  VvTPS35  VvTPS50 | AM461327.2  AM462718.2 | *Vitis vinifera*  *Vitis vinifera* |
| TPS-c  ZmCDs  SITPS40  SITPS41  PpCPS | L37750  JN412074  JN412073  BAF61135 | *Zea mays*  *Solanum lycopersicum*  *Solanum lycopersicum*  *Physcomitrella patens* |
| TPS-d  PsLIMs  AgPHEs  PaMYRs  PaFARs  PaLINs  PaCARs  AgLIMs  AgMYRs | DQ195275.1  AAF61453  AAS47696  AAS47697  AAS47693  AAO73863  AAB70907  AAB71084 | *Picea sitchensis*  *Abies grandis*  *Picea abies*  *Picea abies*  *Picea abies*  *Picea abies*  *Abies grandis*  *Abies grandis* |
| TPS-e/f  SITPS21  SITPS18  SITPS24  CsKS1 | JN412087  JN412088  JN412086  AB045310 | *Solanum lycopersicum*  *Solanum lycopersicum*  *Solanum lycopersicum*  *Cucumis sativus* |
| TPS-g  SITPS39  SITPS37  AmMARs | JN412075  JN412077  AY195609 | *Solanum lycopersicum*  *Solanum lycopersicum*  *Antirrhinum majus* |

Accession numbers of CYP450 proteins used in the phylogenetic analysis:

| Enzyme name | Accession No. | Species |
| --- | --- | --- |
| CYP71  CYP71A1  CYP71A2  CYP71A5  CYP71A32  CYP71BL1  CYP71BL2  CYP71BL3  GAO5  GAO4  GAO3  GAO2  GAO1  CYP71D55  CYP71D20  CYP71D18  CYP71D15  CYP71D13 | P24465.2  Q947B7.1  CAA70575.1  AAL06397.1  F8S1H3.1  F8S1I0.1  G3GBK0.1  ADF43083.1  ADF43082.1  ADF43081.1  ADF43080.1  ADF32078.1  A6YIH8.1  Q94FM7.2  Q9XHE8.1  Q9XHE6.1  Q9XHE7.1 | *Persea americana*  *Mentha x piperita*  *Nepeta racemosa*  *Mentha x piperita*  *Helianthus annuus*  *Lactuca sativa*  *Cichorium intybus*  *Barnadesia spinosa*  *Helianthus annuus*  *Saussurea costus*  *Cichorium intybus*  *Lactuca sativa*  *Hyoscyamus muticus*  *Nicotiana tabacum*  *Mentha spicata*  *Mentha x piperita*  *Mentha x piperita* |
| CYP72  CYP72A1  CYP72A154 | Q05047.1.2  BAL45207.1 | *Catharanthus roseus*  *Glycyrrhiza uralensis* |
| CYP76  CYP76B1  CYP76B6  CYP76B10 | AAK60517.1  Q8VWZ7.1  D1MI46.1 | *Gossypium arboretum*  *Catharanthus roseus*  *Swertia mussotii* |
| CYP88  CYP88A1  CYP88A2  CYP88A3  CYP88A4  CYP88A6  CYP88D6  CYP887 | Q43246.1  AAG41777.1  O23051.1  Q9C5Y2.2  AAO23063.1  B5BSX1.1  AAO23064.1 | *Zea mays*  *Cucurbita maxima*  *Arabidopsis thaliana*  *Arabidopsis thaliana*  *Pisum sativum*  *Glycyrrhiza uralensis*  *Pisum sativum* |
| CYP93  CYP93E1  CYP93E2  CYP93E3 | Q9XHC6.1  ABC59085.1  BAG68930.1 | *Glycine max*  *Medicago truncatula*  *Glycyrrhiza uralensis* |
| CYP97  CYP97A3  CYP97C1 | Q93VK5.1  Q6TBX7.1 | *Arabidopsis thaliana*  *Arabidopsis thaliana* |
| CYP701  CYP701A1  CYP701A3 | AAG41776.1  Q93ZB2.2 | *Cucurbita maxima*  *Arabidopsis thaliana* |
| CYP716  CYP16A12 | ABC59076.1 | *Medicago truncatula* |
| CYP720  CYP720B1 | Q50EK6.1 | *Pinus taeda* |
| CYP725  CYP725A  CYP725A1  CYP725A2  CYP725A3 | Q6WG30.2  Q9AXM6.1  Q8W4T9.1  Q84KI1.1 | *Taxus cuspidate*  *Taxus cuspidate*  *Taxus cuspidate*  *Taxus cuspidate* |
| CYP735  CYP735A1  CYP735A2 | Q9FF18.1  Q9ZW95.1 | *Arabidopsis thaliana*  *Arabidopsis thaliana* |
